# Cleavage of TOM1 by the SARS-CoV-2 main protease NSP5 prevents autophagic degradation of viral envelope

**DOI:** 10.1128/jvi.00434-26

**Published:** 2026-06-12

**Authors:** Qingxiang Zhang, Jingguo Xin, Chunlei Wang, Xue Zhang, Yuan Gao, Wenying Gao, Wenyan Zhang

**Affiliations:** 1 Institute of Virology and AIDS Research, Centre of Infectious Diseases and Pathogen Biology, Key Laboratory of Organ Regeneration and Transplantation of the Ministry of Education, the First Hospital of Jilin University 117971, Changchun, China; 2 Jilin Provincial Key Laboratory on Molecular and Chemical Genetics, the Second Hospital of Jilin University 154454 , Changchun, China; The Ohio State University 2647, Columbus, Ohio, USA

**Keywords:** SARS-CoV-2, NSP5 protease, TOM1, TOLLIP, cleavage

## Abstract

Autophagy plays a critical role in viral replication and the regulation of host immune responses. Although the TOM1-TOLLIP complex has been implicated in immune signaling, cargo trafficking, and endocytosis, its role in viral replication has not been defined. Here, we demonstrate that the SARS-CoV-2 main protease (NSP5) cleaves TOM1 at residue Q354 through its protease activity. This cleavage is also observed with the main proteases of SARS-CoV and MERS-CoV. Importantly, TOM1 overexpression suppresses SARS-CoV-2 replication in HEK293T-hACE2 and Vero cells, while TOM1 knockout via CRISPR-Cas9 significantly enhances viral propagation, indicating that TOM1 functions as a novel restriction factor against SARS-CoV-2 infection. Moreover, various TOM1 orthologs from diverse species, including cattle, bats, monkeys, mice, and ducks, show similar restriction to SARS-CoV-2, but they all are antagonized by NSP5 cleavage. Mechanistically, we found that TOM1 recruits the autophagy receptor TOLLIP to target SARS-CoV-2 envelope (E) protein for autophagic degradation, thereby limiting viral replication. These findings highlight the importance of the TOM1-TOLLIP complex in host defense and discover that SARS-CoV-2 exploits a conserved NSP5-mediated TOM1 cleavage mechanism to evade host antiviral defenses.

**IMPORTANCE:**

Viruses must overcome the body’s natural defenses in order to replicate and spread. One important cellular defense mechanism is autophagy, a process that helps cells remove harmful proteins and pathogens. In this study, we discovered that a host protein called TOM1 acts as a restriction factor that helps limit the replication of SARS-CoV-2, the virus responsible for COVID-19. TOM1 works together with another protein, TOLLIP, to direct envelope proteins to the cell’s degradation system, thereby reducing viral replication. However, SARS-CoV-2 has evolved a strategy to counter this defense. The viral main protease (NSP5) cleaves TOM1, disabling its antiviral activity. This mechanism is conserved among several coronaviruses, including SARS-CoV and MERS-CoV. Our findings reveal a previously unrecognized antiviral role of the TOM1-TOLLIP complex and demonstrate how coronaviruses evade this host defense, providing new insight into virus-host interactions and potential targets for antiviral therapies.

## INTRODUCTION

Severe acute respiratory syndrome coronavirus 2 (SARS-CoV-2), the causative agent of coronavirus disease 2019 (COVID-19), has spread worldwide, profoundly affecting both public health and the global economy (1, 2). Six years into the pandemic, despite the availability of vaccines and therapeutic agents, SARS-CoV-2 remains a major threat due to vaccine hesitancy and the emergence of variants with enhanced immune evasion, replicative fitness, and transmissibility (3, 4). Defining the host-pathogen interactions essential for SARS-CoV-2 replication will improve the understanding of its biology and support the development of host-directed antiviral strategies with broad-spectrum potential (5).

The RNA genome of SARS-CoV-2 contains a methylated cap structure at the 5′ end and a polyadenylated tail (poly(A)) at the 3′ end. The open reading frames ORF1a and ORF1b, located near the 5′ end, occupy approximately two-thirds of the genome and are translated into the polyproteins pp1a and pp1ab (6, 7). These polyproteins are subsequently cleaved by the papain-like protease (PLpro) and the main protease (3CLpro/NSP5) to generate 16 nonstructural proteins, which assemble into the replication-transcription complex (RTC) that drives viral replication and transcription (8, 9). In addition to processing viral polyproteins, NSP5 also cleaves host restriction factors. For example, SARS-CoV-2 suppresses antiviral innate immune signaling by cleaving TAB1 and NLRP12 and evades immune response to cleave RNF20, thereby promoting viral replication (10, 11). In addition to disrupting innate immune signaling, SARS-CoV-2 also manipulates other host defense mechanisms, such as autophagy, to promote viral replication. These findings underscore the need to understand how cellular degradative pathways are regulated during infection (12–14).

Autophagy is a conserved catabolic pathway that degrades macromolecules and organelles via lysosomes to maintain cellular homeostasis and support survival under stress (15, 16). This process requires the coordinated action of autophagy-related proteins, which direct substrates to autophagosomes for degradation. Although initially considered a non-selective process, accumulating evidence indicates that cells can selectively target damaged organelles, protein aggregates, and intracellular pathogens (17, 18). In selective autophagy, specific receptors contain ubiquitin-binding domains (UBD or CUE) and LC3-interacting regions (LIR). These receptors recognize polyubiquitin chains on target proteins through their UBD/CUE domains while simultaneously binding LC3 on the autophagosomal membrane via their LIR domains, thereby delivering cargo to the autophagosome. Fusion of the autophagosome with the lysosome then facilitates degradation (19, 20). Well-characterized selective autophagy receptors include p62/SQSTM1 (sequestosome 1), NBR1 (NBR1 autophagy cargo receptor), OPTN (optineurin), TOLLIP (toll-interacting protein), and BNIP3L/NIX (BCL2-interacting protein 3-like). These receptors act as adaptors linking ubiquitinated substrates to the autophagy machinery and have been extensively studied in diverse biological contexts (21–24). Beyond maintaining homeostasis, selective autophagy receptors play critical roles in antiviral defense by targeting viral components for degradation. For example, NBR1 binds and mediates the autophagic degradation of the porcine deltacoronavirus/PDCoV envelope (E) protein, thereby inhibiting infection (25). Similarly, p62/SQSTM1 targets Zika virus/ZIKV NS3 and NS5 proteins for lysosomal degradation, suppressing viral proliferation (26). NLRC5 suppresses Dengue virus/DENV replication by recruiting TOLLIP to facilitate the autophagic degradation of NS3 (27). Although autophagy is increasingly implicated in viral infection, it remains unclear whether TOM1, in complex with TOLLIP, contributes to the regulation of viral replication.

The TOM1 gene was initially identified in mammalian cells and encodes a cytoplasmic protein that was originally considered an oncogene (28, 29). This protein is widely expressed in various tissues, with particularly high levels in the immune and nervous systems (30, 31). Structurally, TOM1 contains an N-terminal VHS domain, a central GAT domain, and a non-conserved C-terminal region (32). The VHS domain is implicated in endosomal vesicular trafficking, while the GAT domain mediates specific interactions with ubiquitin and TOLLIP (33). Previous studies have shown that TOM1 binding promotes the dissociation of TOLLIP from the endosomal membrane, thereby enabling TOLLIP to engage in cargo transport processes (34–36). Given its role in endosomal trafficking and interaction with TOLLIP, TOM1 represents a potential regulator of autophagy during viral infection, and whether it is targeted by viral proteases as an immune evasion strategy; however, this possibility remains unexplored.

In this study, we demonstrate that the main protease NSP5 of SARS-CoV-2 cleaves human TOM1 and its homologs from multiple species at residue Q354. Correspondingly, TOM1 exerts potential anti-SARS-CoV-2 replication by recruiting the autophagy receptor TOLLIP to facilitate autophagic degradation of the SARS-CoV-2 E protein, although cleavage of TOM1 by NSP5 abolishes its antiviral activity. Collectively, these findings uncover a previously unrecognized mechanism by which TOM1 contributes to host antiviral defense through the recruitment of TOLLIP. Meanwhile, we further provide new mechanistic insights into how coronaviruses subvert host autophagy, highlighting TOM1 as a potential target for broad-spectrum antiviral intervention.

## RESULTS

### Identification of TOM1 as a substrate protein of the SARS-CoV-2 protease NSP5

NSP5, the main protease of SARS-CoV-2, is a critical factor in the viral life cycle, which cleaves the viral polyprotein to release functional proteins that assemble into the replication complex and also suppresses host antiviral responses. To identify the potential NSP5 substrates, we first used mass spectrometry (MS) to screen proteins interacting with NSP5. Considering that 3C-like proteases can cleave and degrade their binding partners, we employed a catalytically inactive NSP5 double mutant (NSP5-DM) as bait in co-immunoprecipitation (co-IP) experiments (Fig. S1A). Compared with the empty vector control, transfection with the inactive NSP5 mutant yielded 84 differentially enriched proteins. We next analyzed the flanking residues (P4–P4′) of cleavage sites in the SARS-CoV-2 polyprotein and previously reported host substrates (Fig. S1B). As shown in Fig. S1C, NSP5 specifically cleaves at the L (P2)-Q (P1)-S/A (P1′) peptide bond. Based on this motif, we identified 15 interacting proteins containing the sequence (Fig. S1D). MYO6, whose cleavage motif is located at its C-terminus, was excluded from further analysis. To evaluate protease accessibility, we performed structural simulations of the remaining 14 proteins using AlphaFold3 and assessed structural flexibility with predicted aligned error (PAE) heatmaps. The results indicated that only five proteins, TOM1, SQSTM1, PSMC2, EIF3L, and AURKA, had exposed cleavage sites, while the motifs in the other proteins were buried and inaccessible to the protease active site (Fig. S2).

**Fig 1:**
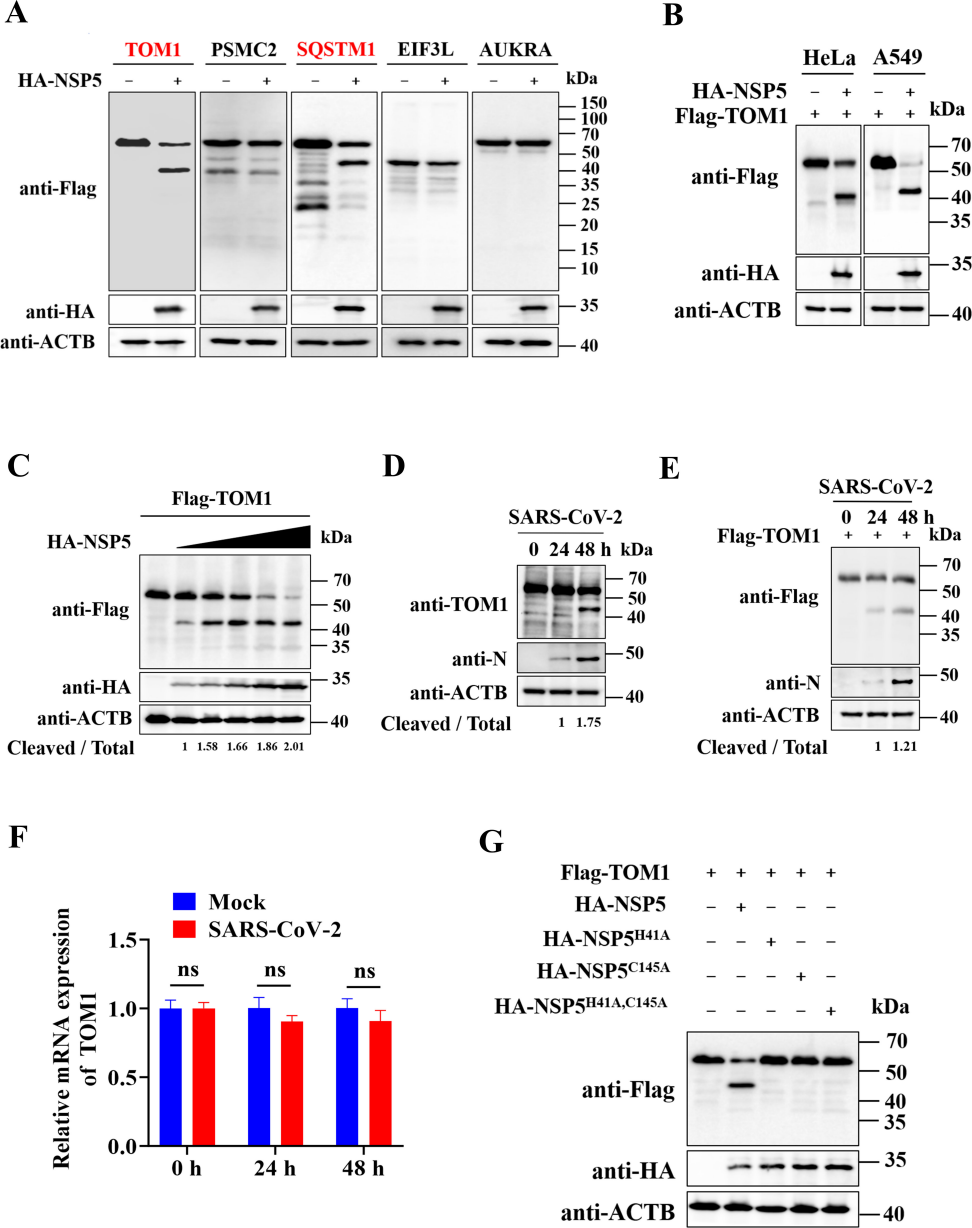
Identification of TOM1 as a substrate protein for the SARS-CoV-2 protease NSP5. (**A**) HEK293T cells were transfected with plasmids encoding Flag-tagged host proteins, along with empty vector or HA-NSP5; after 24 h, cell lysates were subjected to immunoblot (IB) analysis using the indicated antibodies. (**B**) A549 and HeLa cells were co-transfected with plasmids encoding Flag-TOM1 and HA-SARS-CoV-2 NSP5; at 24 h post-transfection, the cells were harvested for IB. (**C**) HEK293T cells were co-transfected with Flag-TOM1 and varying doses of HA-NSP5. After 24 h, cell lysates were prepared for IB. (**D**) HEK293T-hACE2 cells were infected with SARS-CoV-2 and harvested at 24 and 48 h post-infection (hpi) to detect TOM1 expression by IB. (**E**) HEK293T-hACE2 cells were transfected with Flag-TOM1 for 24 h, and then, the cells were infected with SARS-CoV-2 for the indicated times. The cells were harvested for IB. (**F**) TOM1 mRNA levels were examined by RT-qPCR in SARS-CoV-2-infected HEK293T-hACE2 cells (ns, no significant difference). (**G**) HEK293T cells were co-transfected with plasmids encoding Flag-TOM1 and HA-tagged NSP5 wild-type (WT) or its protease-defective mutant (H41A, C144A, or H41A, C144A). At 24 h post-transfection, the cells were harvested and analyzed by IB.

We next examined the cleavage of these five candidate substrates by NSP5, and the results showed that NSP5 efficiently cleaved TOM1 and SQSTM1 (Fig. 1A). Given that cleavage of SQSTM1 by NSP5 has been reported previously, subsequent studies focused on TOM1 (37). To exclude cell type-specific effects, TOM1 cleavage was confirmed in both HeLa and A549 cells (Fig. 1B). Notably, increasing NSP5 expression induced dose-dependent TOM1 cleavage (Fig. 1C). To assess whether endogenous TOM1 is cleaved during SARS-CoV-2 infection, we analyzed TOM1 expression in SARS-CoV-2-infected 293T-hACE2 cells. Viral infection significantly reduced full-length TOM1 protein levels and generated a 42-kDa fragment recognized by an N-terminal antibody (Fig. 1D). Similarly, overexpressed TOM1 exhibited a cleavage band upon infection (Fig. 1E). Finally, to determine whether the reduction in TOM1 protein was due to transcriptional downregulation, we measured TOM1 mRNA levels by RT-qPCR. As shown in Fig. 1F, TOM1 mRNA level remained stable after infection, indicating that the reduction in protein levels is caused by proteolytic cleavage rather than decreased gene expression. To investigate whether the protease activity of NSP5 is required for TOM1 cleavage, HEK293T cells were co-transfected with Flag-TOM1 and either wild-type NSP5 or three protease-deficient mutants (NSP5^H41A^, NSP5^C145A^, or NSP5^H41A,C145A^). TOM1 cleavage was observed only in cells expressing wild-type NSP5, confirming that its protease activity is essential for TOM1 processing (Fig. 1G). Collectively, these results demonstrate that SARS-CoV-2 relies specifically on the protease activity of NSP5 to cleave TOM1.

### SARS-CoV-2 NSP5 cleaves TOM1 at residue Q354

**Fig 2:**
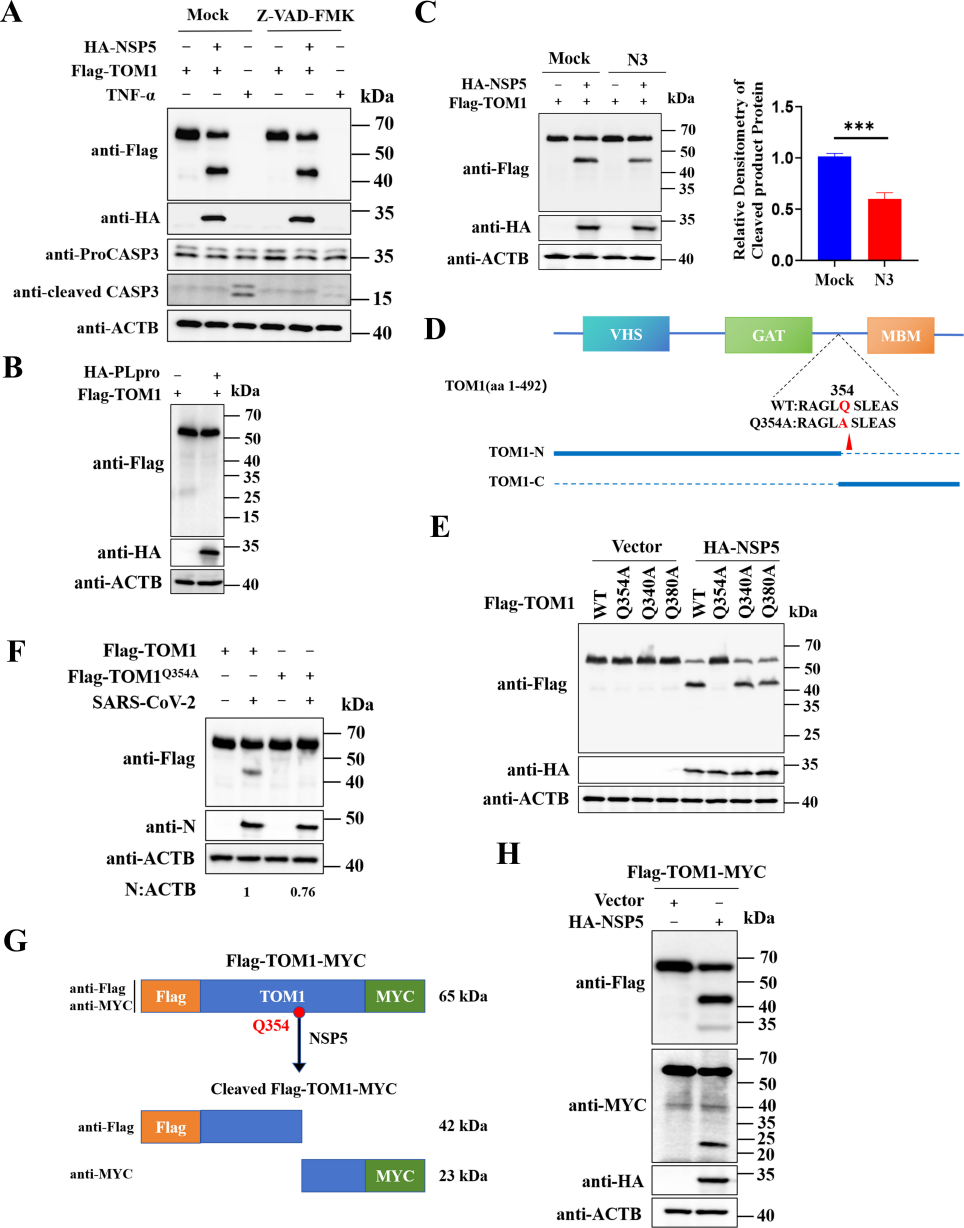
SARS-CoV-2 NSP5 cleaves TOM1 at residue Q354. (**A**) HEK293T cells were co-transfected with HA-NSP5 along with Flag-TOM1, followed by treatment with Z-VAD-FMK (20 μM); cell lysates were prepared for IB. TNF-α (50 ng/mL) was used as a positive control to verify the inhibition of CASP3 activity by Z-VAD-FMK. (**B**) HEK293T cells were co-transfected with HA-PLpro along with Flag-TOM1; then, cell lysates were analyzed by IB. (**C**) HEK293T cells were transfected with HA-NSP5 and TOM1 for 24 h. Subsequently, the cells were treated with N3 (10 μM) or DMSO for 12 h, followed by IB analysis. The intensities of cleavage products were normalized, relative to non-cleavage, based on three independent experiments. (**D**) Schematic representation of the TOM1 domain and the predicted cleavage site Q354 by NSP5. (**E**) HEK293T cells were co-transfected with HA-NSP5 and Flag-TOM1 or its mutants. After 24 h, cell lysates were subjected to IB. (**F**) HEK293T-hACE2 cells were transfected with WT-TOM1 or the TOM1-Q354A mutant and subsequently infected with SARS-CoV-2. At 48 hpi, cell lysates were analyzed by IB. (**G**) Schematic representation of Flag-TOM1-MYC cleavage mediated by the NSP5. (**H**) HEK293T cells were co-transfected with Flag-TOM1-MYC and HA-NSP5. At 24 h post-transfection, the cells were harvested for IB. (ns, not significant; *P < 0.05; **P < 0.01; ***P < 0.001; ****P < 0.0001).

To determine whether NSP5-induced TOM1 cleavage is caspase-dependent, HEK293T cells were co-transfected with Flag-TOM1 and HA-NSP5, with a subset of cells treated with the caspase inhibitor Z-VAD-FMK. As shown in Fig. 2A, Z-VAD-FMK treatment did not affect TOM1 cleavage but effectively inhibited the positive control, TNF-α-induced caspase 3 (CASP3) activation, indicating that NSP5 cleaves TOM1 independently of the caspase pathway. As SARS-CoV-2 also encodes a papain-like protease (PLpro), we next tested its potential involvement. Co-transfected Flag-TOM1 and HA-PLpro did not lead to TOM1 cleavage (Fig. 2B). In contrast, treatment with N3, an inhibitor of SARS-CoV-2 3CLpro/NSP5, markedly reduced NSP5-mediated TOM1 cleavage (Fig. 2C).

Based on the NSP5 cleavage consensus motif (Fig. 1B), glutamine at position 354 (Q354) of TOM1 was predicted as a potential cleavage site (Fig. 2D). Considering the observed molecular weight of the N-terminal cleavage fragment (42-kDa), Q340, Q354, and Q380 were evaluated as potential candidate sites. To define the exact cleavage site, three TOM1 mutants (Q340A, Q354A, and Q380A) were generated by substituting the respective glutamine (Q) residues with alanine (A). These constructs were co-expressed with NSP5 in HEK293T cells. Western blotting analysis revealed that the TOM1^Q354A^ mutant was resistant to NSP5-mediated cleavage, whereas TOM1^Q340A^ and TOM1^Q380A^ remained susceptible (Fig. 2E). Consistently, during SARS-CoV-2 infection, a cleavage product was observed in 293T-hACE2 cells expressing wild-type TOM1, but not in cells transfected with TOM^Q354A^ (Fig. 2F). Together, these findings demonstrate that SARS-CoV-2 NSP5 specifically cleaves TOM1 at Q354. To further validate cleavage at Q354, we constructed a dual-tagged TOM1 plasmid (Flag-TOM1-MYC) and co-transfected it with NSP5 into HEK293T cells. Western blotting with anti-Flag and anti-MYC antibodies detected cleavage products 42-kDa (N-terminal fragment) and 23-kDa (C-terminal fragment), respectively (Fig. 2G and H). The sizes of these fragments are consistent with proteolysis occurring at Q354.

### TOM1 inhibits SARS-CoV-2 replication

**Fig 3:**
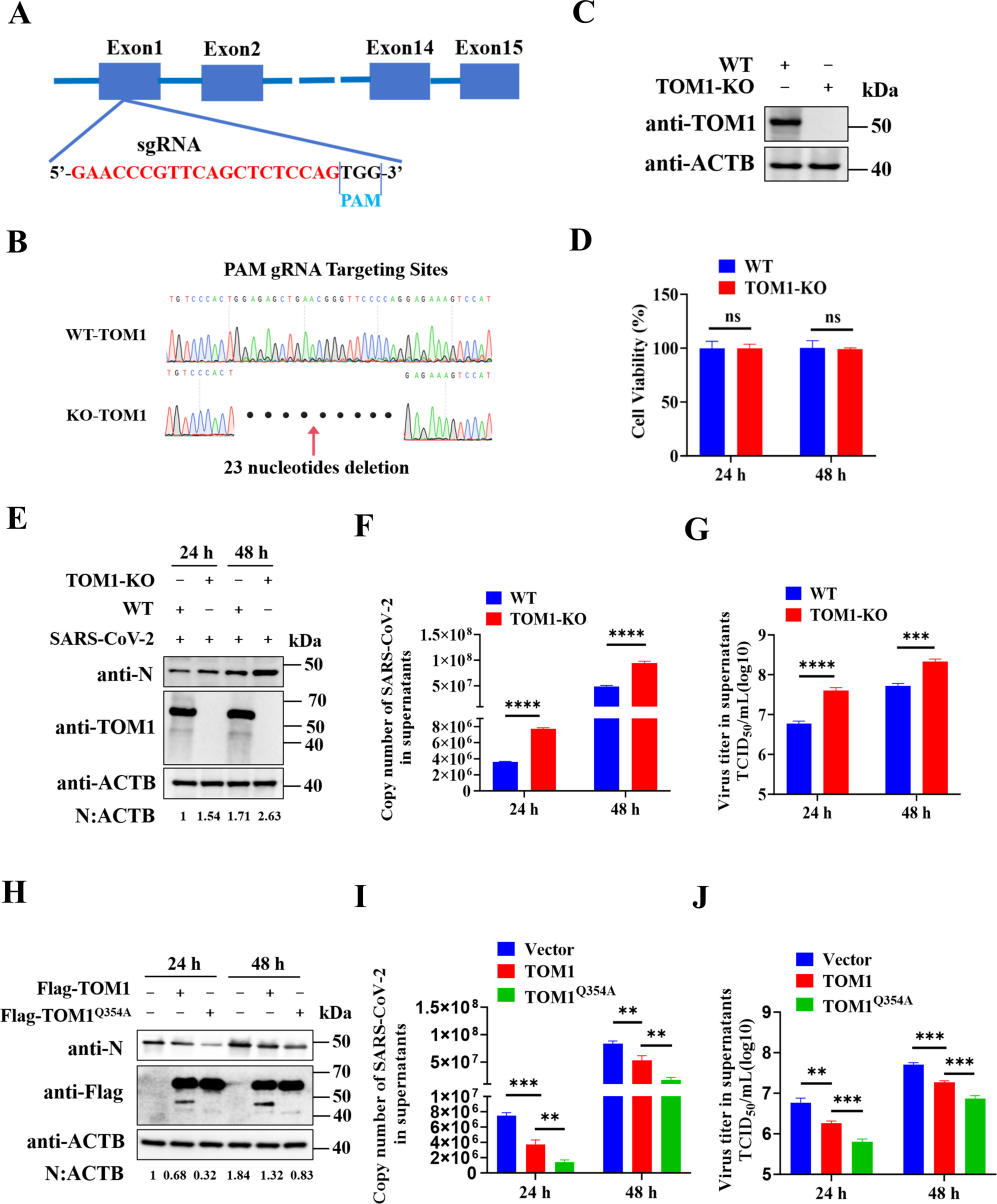
TOM1 inhibits SARS-CoV-2 replication. (**A**) Schematic representation of the TOM1 knockout strategy. (**B and C**) Validation of TOM1 knockout in HEK293T-hACE2 cells by DNA sequencing (**B**) and IB (**C**). (**D**) Cell viability of WT and *TOM1-KO* HEK293T-hACE2 cell lines. (**E through G**) WT and *TOM1-KO* HEK293T-hACE2 cell lines were infected with SARS-CoV-2; the cells were collected at the indicated times for IB (**E**). The harvested supernatants were used for RT-qPCR (**F**) and TCID_50_ (**G**) assays. (**H through J**) HEK293T-hACE2 cells were transfected with Flag-TOM1, Flag-TOM1^Q354A^, or an empty vector, followed by SARS-CoV-2 infection. At 24 and 48 hpi, the cells were collected for IB (H), the harvested supernatants were used for RT-qPCR (I) and TCID_50_ (J) assays. Data are presented as mean ± SEM from three independent experiments. (ns, no significant; *P < 0.05; **P < 0.01; ***P < 0.001; ****P < 0.0001).

Building on the finding that SARS-CoV-2 NSP5 cleaves TOM1, we next investigated whether TOM1 influences viral replication. A TOM1-knockout (*TOM1-KO*) 293T-hACE2 cell line was established using CRISPR-Cas9. Sequencing analysis confirmed a 23-nucleotide deletion in the first exon of TOM1 (Fig. 3A and B), and western blotting verified complete loss of TOM1 protein (Fig. 3C). Cell viability was unaffected by TOM1 deletion, as determined by CCK-8 assay (Fig. 3D). We next assessed the impact of *TOM1-KO* on SARS-CoV-2 replication. *TOM1-KO* cells exhibited increased viral replication in both cells and supernatants, with viral titers significantly elevated in the supernatant (Fig. 3E through G). Conversely, overexpression of TOM1 in 293T-hACE2 cells suppressed SARS-CoV-2 replication, as evidenced by reduced N gene expression in both cells and supernatants, and by lower viral titers at 24 or 48 h post-infection (Fig. 3H through J). Notably, the TOM1^Q354A^ mutant, which resisted NSP5-mediated cleavage, exerted a stronger antiviral effect than wild-type TOM1 (Fig. 3H through J). Parallel experiments in Vero cells confirmed these observations: TOM1 knockout enhanced viral replication (Fig. S3A through F), whereas TOM1 overexpression restricted it (Fig. S3G through I).

### TOM1 targets SARS-CoV-2 E protein for autophagic degradation

**Fig 4:**
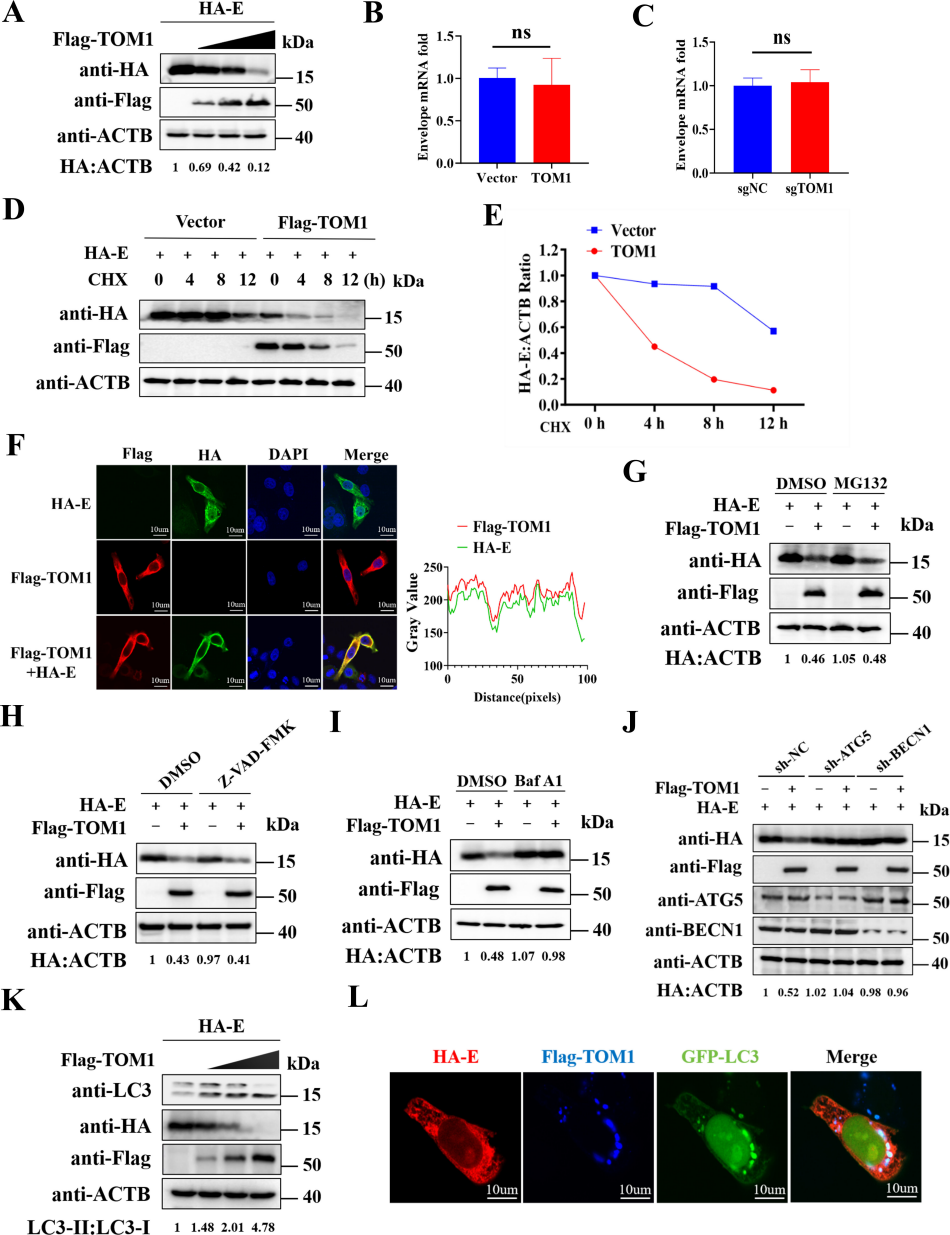
TOM1 targets SARS-CoV-2 E protein for autophagic degradation. (**A**) HEK293T cells were transfected with increasing amounts of Flag-TOM1 together with HA-E protein. IB analysis of protein with the indicated antibodies. (**B and C**) RT-qPCR quantification of E mRNA in HEK293T cells transfected with HA-E and either Flag-TOM1 or *TOM1-KO* (ns, no significant). (**D**) HEK293T cells were transfected with Flag-TOM1 and HA-E. At 28 h post-transfection, the cells were collected at the indicated time points to measure E protein levels by IB after 100 μg/mL CHX treatment. (**E**) Quantification of E protein levels from panel D, normalized to ACTB. (**F**) Co-localization of TOM1 and E was examined in HeLa cells transfected with Flag-TOM1 and HA-E for 28 h. Cell nuclei were stained with DAPI. Scale bars: 10 µm. The line graphs on the right present the quantitative analysis of protein colocalization performed using Image J. (**G through I**) HEK293T cells were co-transfected with Flag-TOM1 and HA-E plasmids, subsequently treated with MG132 (10 µM) (G), Z-VAD-FMK (50 µM) (H), or Bafilomycin A1 (10 μM) (I) for 12 h before harvesting, and then, the cell lysates were analyzed by IB. (**J**) IB analysis of protein from HEK293T cells co-transfected with Flag-TOM1 and HA-E, together with shATG5 or shBECN1 for 28 h. (**K**) HEK293T cells were transfected with increasing amounts of Flag-TOM1 together with HA-E. IB analysis of protein with the indicated antibodies. (**L**) HeLa cells co-transfected with Flag-TOM1, GFP-LC3B, and HA-E for 28 h were fixed, immunostained with anti-Flag and anti-HA antibodies, and examined by confocal microscopy. Scale bar: 10 µm.

TOM1 has been reported to recruit the autophagy receptor TOLLIP to form a cargo-transport complex (35). As a selective autophagy receptor, TOLLIP contributes to antiviral defense by directing viral proteins for degradation (38). Therefore, we hypothesized that TOM1 restricts SARS-CoV-2 replication by recruiting TOLLIP to mediate the autophagic degradation of SARS-CoV-2 proteins. To test this, we first examined whether TOM1 interacts with the four structural proteins. Co-IP assays revealed that TOM1 bound to the E and membrane (M) proteins (Fig. S4A and B), but not the spike (S) or nucleocapsid (N) proteins (Fig. S4C and D). To assess whether TOM1 promotes degradation of E and M proteins, cells were co-transfected with plasmids encoding E or M proteins, together with increasing amounts of TOM1. The results showed that TOM1 reduced E protein levels in a dose-dependent manner (Fig. 4A) but had no effect on M (Fig. S4E). Importantly, TOM1 overexpression or knockout did not alter E mRNA levels (Fig. 4B and C), excluding transcriptional regulation. Cycloheximide (CHX) chase assays further confirmed that TOM1 accelerates E protein degradation (Fig. 4D and E). TOM1 also induced the degradation of all tested SARS-CoV-2 E protein variants (Fig. S4F). Confocal microscopy revealed cytoplasmic co-localization of TOM1 and E (Fig. 4F). Collectively, these findings indicate that TOM1 selectively targets the E protein for degradation.

In eukaryotes, protein degradation occurs mainly through apoptosis, the ubiquitin-proteasome system (UPS), and the autophagy-lysosome pathway (39). To determine which pathway mediates TOM1-dependent E degradation, we treated HEK293T cells co-expressing Flag-TOM1 and HA-E with pathway-specific inhibitors. The results showed that the autophagy inhibitor Bafilomycin A1 (Baf A1) restored E protein degradation, whereas the apoptosis inhibitor Z-VAD-FMK and the proteasome inhibitor MG132 had no effect (Fig. 4G through I). Consistent results were obtained with two additional autophagy inhibitors, 3-methyladenine (3-MA) and vinblastine (Fig. S4G and H). Furthermore, knockdown of the autophagy regulators ATG5 or BECN1 abolished the TOM1-induced reduction of E protein levels (Fig. 4J). In contrast, autophagy induction with the mTOR inhibitors rapamycin and Torin1 enhanced TOM1-mediated E degradation (Fig. S4I and J). Co-expression of Flag-TOM1 and HA-E not only promoted E protein degradation but also enhanced the conversion of LC3-I to LC3-II in a dose-dependent manner, suggesting that TOM1 enhances autophagy (Fig. 4K). Further immunofluorescence co-localization analysis showed that TOM1 and E co-localized with LC3B-labeled autophagosomes (Fig. 4L). Together, these results demonstrate that TOM1 promotes E protein degradation via the autophagy pathway.

### TOM1 recruits TOLLIP as an autophagy receptor to facilitate the degradation of the E protein

**Fig 5:**
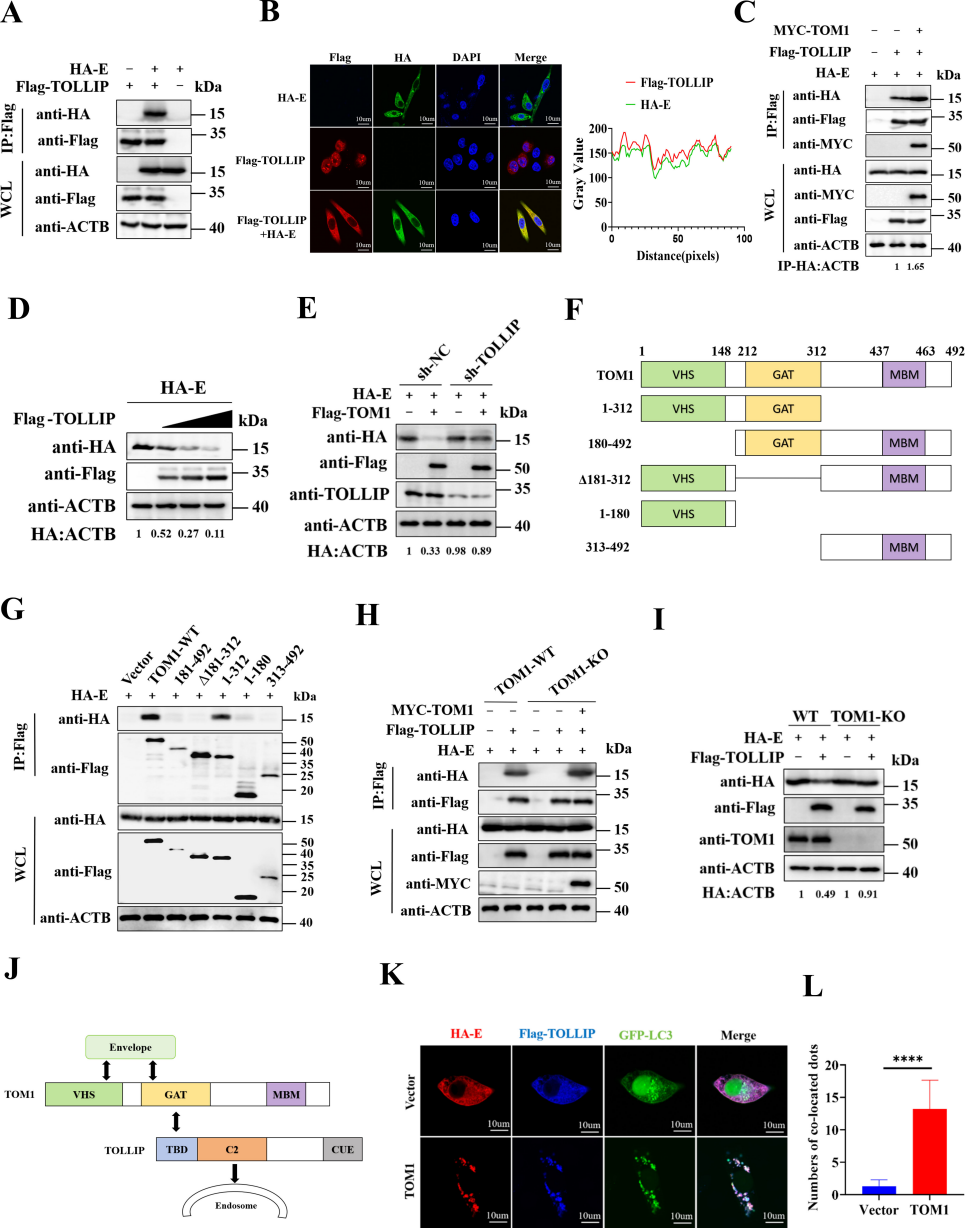
TOM1 recruits TOLLIP as an autophagy receptor to facilitate the degradation of the E protein. (**A**) HEK293T cells were co-transfected with Flag-TOLLIP and HA-E plasmids, and then 10 µM Bafilomycin A1 was added for 12 h prior to harvest. After 48 h, cell lysates were co-immunoprecipitated using anti-Flag antibody, followed by IB analysis. (**B**) Co-localization of TOLLIP and E was examined in HeLa cells transfected with Flag-TOLLIP and HA-E for 28 h, and then 10 µM Bafilomycin A1 was added for 12 h prior to harvest. Cell nuclei were stained with DAPI. Scale bars: 10 µm. The line graphs on the right present the quantitative analysis of protein colocalization performed using Image J. (**C**) HEK293T cells were co-transfected with Flag-TOLLIP, HA-E, and MYC-TOM1 plasmids, and then 10 µM Bafilomycin A1 was added for 12 h to avoid the E degradation prior to harvest. After 48 h, cell lysates were co-immunoprecipitated using anti-Flag antibody, followed by IB analysis. (**D**) IB analysis of lysates of HEK293T cells transfected with increasing amounts of Flag-TOLLIP together with HA-E. (**E**) IB analysis of lysates of HEK293T cells co-transfected with HA-E and shTOLLIP, together with or without Flag-TOM1 for 48 h. (**F**) Schematic representation of TOM1 domains. (**G**) HEK293T cells were co-transfected with HA-E or its deletion mutants along with Flag-TOM1 and then 10 µM Bafilomycin A1 was added for 12 h to avoid the E degradation prior to harvest. After 48 h, cell lysates were co-immunoprecipitated using anti-Flag antibody, followed by IB analysis. (**H**) WT and *TOM1-KO* HEK293T cell lines were transfected with the indicated plasmids expressing HA-E, Flag-TOLLIP, or MYC-TOM1 and then 10 µM Bafilomycin A1 was added for 12 h to avoid the E degradation prior to harvest. After 48 h, cell lysates were co-immunoprecipitated using anti-Flag antibody, followed by IB analysis. (**I**) Flag-TOLLIP and HA-E were co-transfected into WT and *TOM1-KO* HEK293T cells, and IB analysis was carried for proteins with the indicated antibodies. (**J**) A schematic working model of E protein autophagic degradation mediated by the TOM1-TOLLIP complex. (**K**) HeLa cells were co-transfected with HA-E, Flag-TOLLIP, and GFP-LC3B, together with MYC-TOM1 or an empty vector, for 28 h. Scale bars: 10 µm. (**L**) The number of E (red), TOLLIP (blue), and LC3 (green) colocalized puncta in cells was quantified by counting, with eight cells analyzed for both the vector control and TOM1 groups.

Autophagy receptors are known to play critical roles in substrate recognition and degradation through selective autophagy (40). Because TOM1 is not an autophagy receptor, we hypothesized that it may function as a bridging molecule linking the E protein to the autophagy receptor TOLLIP, thereby facilitating its autophagic degradation. Co-IP assays revealed an interaction between TOM1 and TOLLIP (Fig. S5A). Furthermore, we demonstrated that TOLLIP interacted with E (Fig. 5A). Confocal microscopy showed that when expressed alone, TOLLIP localized to the nucleus, with a small portion in the cytoplasm, similar to previous reports (41–43). However, upon co-expression with E, it redistributed to the cytoplasm (Fig. 5B). Overexpression of TOM1 further enhanced the interaction between TOLLIP and E (Fig. 5C). To determine whether TOLLIP mediates TOM1-induced E protein degradation, we co-transfected cells with E and increasing amounts of TOLLIP. TOLLIP promoted E degradation in a dose-dependent manner (Fig. 5D), whereas TOLLIP knockdown significantly attenuated TOM1-induced E protein degradation (Fig. 5E). Consistently, CHX chase assays showed that E degradation was markedly slower in TOLLIP-knockdown cells than in controls (Fig. S5B and C). Given TOLLIP’s ability to mediate E protein degradation, we hypothesized that TOLLIP would similarly inhibit SARS-CoV-2 replication. Unexpectedly, TOLLIP expression alone modestly enhanced SARS-CoV-2 replication, whereas co-overexpression of TOLLIP and TOM1 markedly suppressed viral proliferation (Fig. S5D through F). We propose that TOLLIP-mediated promotion of SARS-CoV-2 might because TOLLIP antagonizes the innate immune response by targeting the RNA-sensing pathway, a notion consistent with a recent report (44).

Autophagy receptors typically mediate selective autophagy through ubiquitin (Ub)-dependent mechanisms. The CUE domain of TOLLIP specifically binds ubiquitin chains and recruits ubiquitinated substrates to autolysosomes. To examine whether TOM1 influences E ubiquitination, we assessed E polyubiquitination. However, neither TOM1 overexpression nor knockout had any effect (Fig. S5G and H). Consistently, a TOLLIP mutant lacking the CUE domain (TOLLIPΔCUE) still bound E (Fig. S5I), and TOM1 was able to degrade lysine-mutated forms of E (Fig. S5J), indicating that TOLLIP-mediated E degradation is independent of ubiquitination.

To elucidate how the TOM1-TOLLIP complex regulates the autophagic degradation of the E protein, we generated a series of TOM1 truncation mutants based on its structural domains (Fig. 5F). Co-IP assays demonstrated that the VHS and GAT domains are essential for the interaction between TOM1 and the E protein (Fig. 5G). Moreover, TOLLIP failed to associate with the E protein in TOM1-knockout cells, whereas rescue expression of TOM1 restored a robust interaction between TOLLIP and the E protein (Fig. 5H). Consistently, we observed that in *TOM1-KO* cells, TOLLIP is unable to degrade the E protein (Fig. 5I). Based on these findings, we propose a model in which TOM1 binds the E protein through its VHS and GAT domains while simultaneously interacting with TOLLIP via its GAT domain; TOLLIP then delivers the E protein to the autophagosome through its C2 domain (Fig. 5J). Supporting this notion, confocal microscopy revealed that TOM1 overexpression enhanced co-localization of the E and TOLLIP with LC3B-marked autophagosomes (Fig. 5K and L).

### Cleavage of TOM1 abrogated its antiviral activity

**Fig 6:**
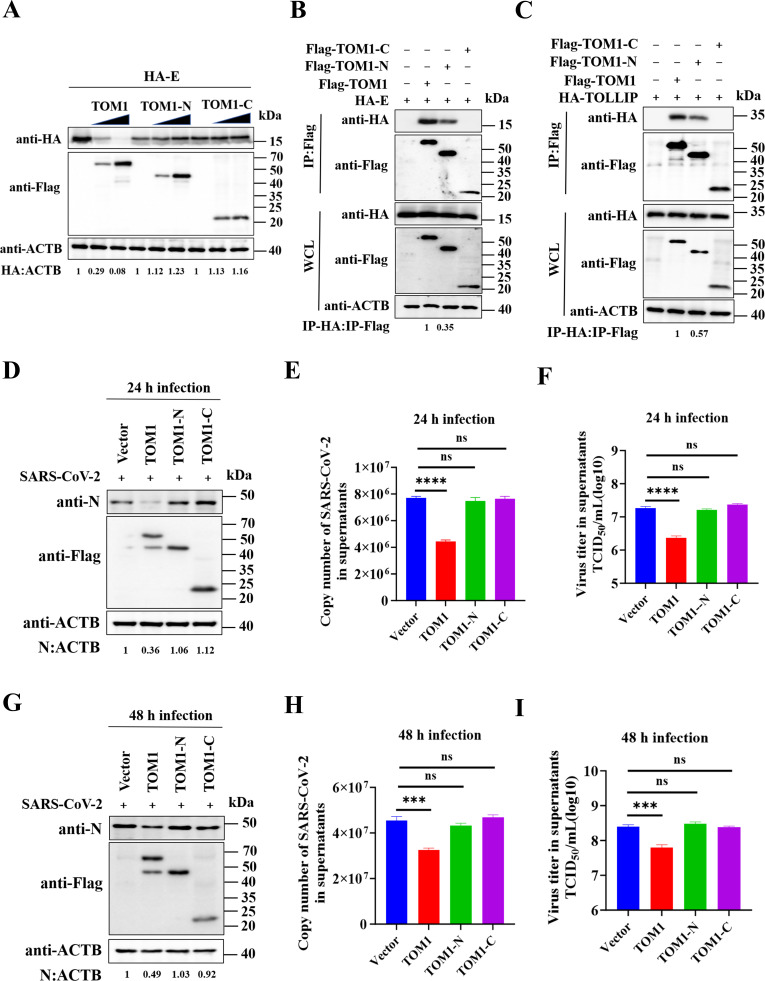
Cleavage of TOM1 abrogates its antiviral activity. (**A**) HEK293T cells were co-transfected with HA-E along with increasing amounts of Flag-TOM1, Flag-TOM1-N, or Flag-TOM1-C, followed by IB analysis. (**B**) HEK293T cells were co-transfected with empty vector, Flag-TOM1, Flag-TOM1-N, or Flag-TOM1-C, together with HA-E, then added 10 µM Bafilomycin A1 for 12 h to avoid the E degradation prior to harvest. After 48 h, cell lysates were co-immunoprecipitated using anti-Flag antibody, followed by IB analysis. (**C**) HEK293T cells were co-transfected with empty vector, Flag-TOM1, Flag-TOM1-N, or Flag-TOM1-C, together with HA-TOLLIP. After 48 h, cell lysates were co-immunoprecipitated using anti-Flag antibody, followed by IB analysis. (**D through I**) HEK293T-hACE2 cells were transfected with Flag-TOM1, Flag-TOM1-N, Flag-TOM1-C, or an empty vector, followed by SARS-CoV-2 infection. At 24 or 48 hpi, the cells were collected for IB analysis (D and G), and the harvested supernatants were used for RT-qPCR (E and H) and TCID_50_ (F and I) assays. Data were presented as mean ± SEM from three independent experiments. (ns, no significant; *P < 0.05; **P < 0.01; ***P < 0.001; ****P < 0.0001).

We next examined the effect of TOM1 cleavage on degradation of the SARS-CoV-2 E protein. HEK293T cells were co-transfected with a plasmid expressing E together with increasing amounts of wild-type TOM1 (TOM1-WT), the N-terminal fragment (TOM1-N), or the C-terminal fragment (TOM1-C). A dose-dependent reduction in E expression was observed in TOM1-WT-expressing cells, whereas no effect was detected with TOM1-N or TOM1-C (Fig. 6A). These results suggest that TOM1 cleavage impairs its ability to mediate E degradation. Consistently, co-IP assays showed that TOM1-N displayed markedly reduced binding to E protein, while TOM1-C completely lost binding capacity (Fig. 6B), indicating that proteolytic cleavage disrupts the interaction between TOM1 and E, thereby impairing TOM1-mediated degradation. Furthermore, co-IP assays revealed that TOM1-N retained a weak interaction with TOLLIP, whereas TOM1-C completely lost its binding capability (Fig. 6C). These findings indicate that 3C-mediated cleavage of TOM1 disrupts the formation of the TOM1-TOLLIP complex. To further assess whether NSP5-mediated cleavage abolishes the antiviral activity of TOM1, HEK293T-hACE2 cells were transfected with plasmids encoding TOM1-WT, TOM1-N, TOM1-C, or an empty vector control, followed by SARS-CoV-2 infection. Cells expressing TOM1-WT displayed significantly reduced SARS-CoV-2 replication within cells and supernatants, as well as decreased viral titers in the supernatant at 24 or 48 h post-infection. In contrast, TOM1-N and TOM1-C failed to reduce SARS-CoV-2 N expression or viral load compared with the empty vector control (Fig. 6D through I). These findings demonstrate that NSP5-mediated cleavage attenuates the antiviral function of TOM1, underscoring the importance of full-length TOM1 in restricting SARS-CoV-2 infection.

### TOM1 is a common substrate of different coronaviruses’ NSP5

**Fig 7:**
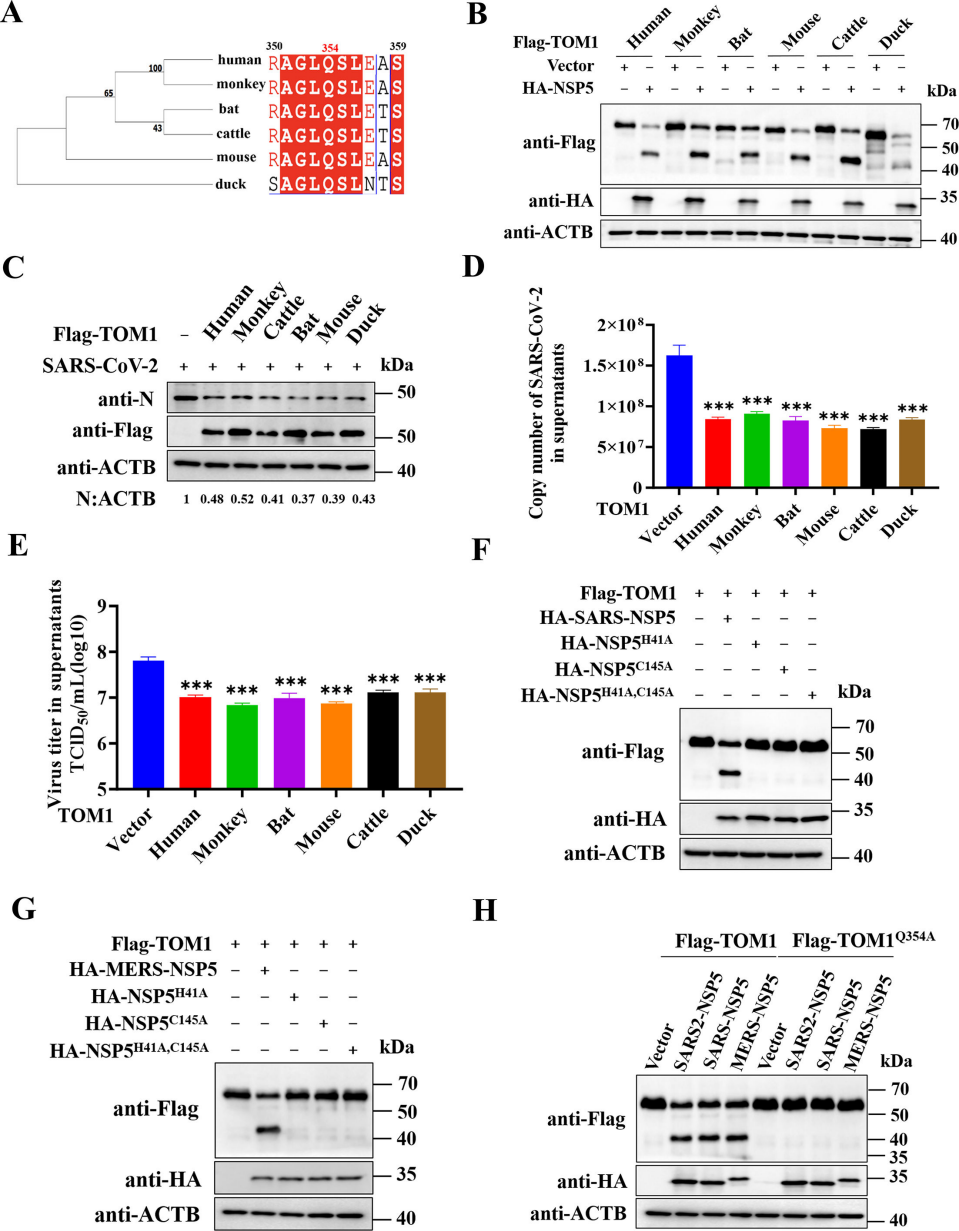
TOM1 is a common substrate of different coronaviruses’ NSP5. (**A**) A phylogenetic tree of TOM1 orthologs was generated using the neighbor-joining method in MEGA11, based on protein sequences surrounding the NSP5 cleavage site from humans, monkeys, mice, cattle, bats, and ducks. (**B**) HEK293T cells were transfected with Flag-tagged TOM1 from the indicated species, along with empty vector or HA-NSP5, followed by IB analysis. (**C through E**) HEK293T-hACE2 cells were transfected with Flag-tagged TOM1 from the indicated species, along with the empty vector, followed by SARS-CoV-2 infection. At 48 hpi, the cells were collected for IB (C), the harvested supernatants were used for RT-qPCR (D) and TCID_50_ (E) assays. Data were presented as mean ± SEM from three independent experiments. (**F and G**) HEK293T cells were transfected with Flag-TOM1 and plasmids encoding HA-tagged NSP5 or their catalytically inactive mutants from SARS-CoV (F) and MERS-CoV (G). After 24 h, cells were lysed and analyzed by IB. (**H**) HEK-293T cells were co-transfected with either wild-type Flag-TOM1 or a mutant (Flag-TOM1^Q354A^), together with the plasmids expressing NSP5s from SARS-CoV--2, SARS-CoV, and MERS-CoV. After 24 h, the cell lysates were collected and analyzed by IB. (ns, not significant; *P < 0.05; **P < 0.01; ***P < 0.001; ****P < 0.0001).

To assess whether NSP5-mediated cleavage of TOM1 is evolutionarily conserved, we performed phylogenetic analyses of TOM1 proteins from mammalian and avian species. The residues at the cleavage site were conserved across humans, monkeys, cattle, bats, mice, and ducks (Fig. 7A). To examine whether NSP5 can cleave TOM1 orthologs from different species, we co-expressed NSP5 with TOM1 orthologs from mammals and birds. NSP5 cleaved all TOM1 orthologs, including those from humans, monkeys, cattle, bats, mice, and ducks (Fig. 7B). To determine whether TOM1 orthologs suppress SARS-CoV-2 replication, we overexpressed TOM1 from various species in HEK293T-hACE2 cells and infected them with SARS-CoV-2. Overexpression of all TOM1 orthologs reduced SARS-CoV-2 N protein expression, N gene mRNA levels in supernatant, and viral titers (Fig. 7C through E). These results indicate that the antagonistic interaction between SARS-CoV-2 NSP5 and TOM1 is evolutionarily conserved. We next examined whether this proteolytic activity is shared by NSP5 proteases of other coronaviruses. The NSP5 proteins of SARS-CoV and MERS-CoV share high similarity with SARS-CoV-2 NSP5, particularly within their catalytic domains. Co-expression assays showed that wild-type, but not protease-deficient mutants, of SARS-CoV or MERS-CoV NSP5 cleaved human TOM1 (Fig. 7F and G). Furthermore, both SARS-CoV and MERS-CoV NSP5 cleaved TOM1 at the same site (Fig. 7H). These findings demonstrate that SARS-CoV, MERS-CoV, and SARS-CoV-2 all cleave TOM1 at Q354 through their protease activities.

## DISCUSSION

**Fig 8:**
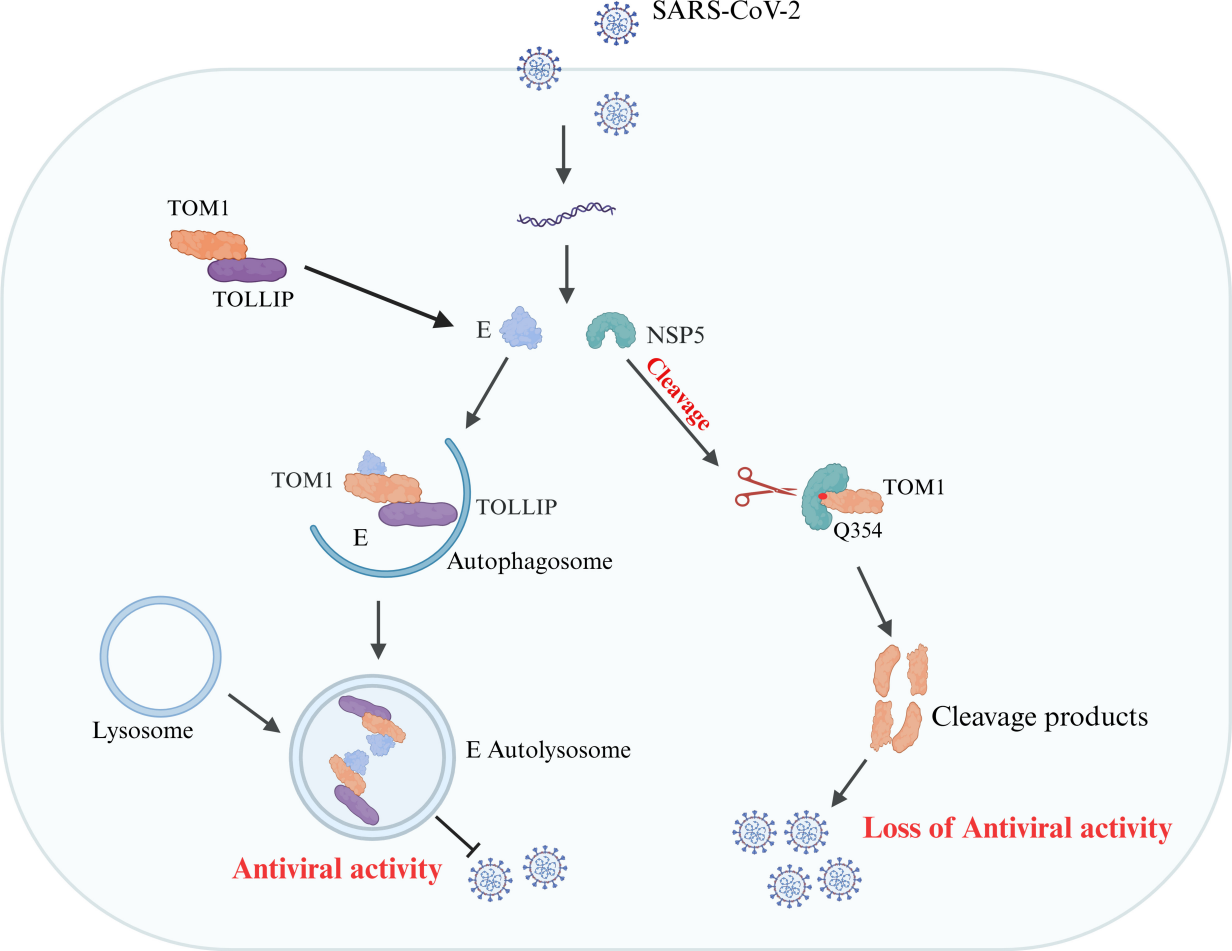
Proposed model depicting the antagonism of TOM1-mediated antiviral activity by coronavirus NSP5.

Autophagy is increasingly recognized as a critical component of host defense against viral infection (45, 46). Here, we identify TOM1 as a novel regulator of autophagy that restricts SARS-CoV-2 replication by recruiting the autophagy receptor TOLLIP to target the viral E protein for lysosomal degradation. Importantly, SARS-CoV-2 counteracts this defense through NSP5-mediated cleavage of TOM1 at Q354, thereby abrogating its antiviral activity (Fig. 8).

Coronaviruses encode proteases that counteract host defenses by targeting essential antiviral proteins (47–49). Consistent with prior reports that SARS-CoV-2 NSP5 cleaves SQSTM1/p62 (37), NLRP12, and TAB1 to impair innate immunity and promote viral infection (10), our work demonstrates that NSP5 also cleaves TOM1 at a conserved Q354 site. This proteolytic event abolishes TOM1’s ability to recruit TOLLIP and mediate E protein degradation, thereby neutralizing its antiviral activity. Importantly, NSP5 orthologs from SARS-CoV and MERS-CoV cleave TOM1 at the same site, highlighting an evolutionarily conserved mechanism by which coronaviruses antagonize TOM1-mediated autophagy. These observations place TOM1 alongside other NSP5 substrates as critical nodes of host defense that are selectively targeted for viral immune evasion.

Autophagy receptors such as SQSTM1, NBR1, and TOLLIP have been shown to target viral proteins for lysosomal degradation, thereby restricting replication of diverse viruses including ZIKV, PDCoV, and Duck Tembusu Virus/DTMUV (25, 26, 38). Our findings extend this paradigm by identifying TOLLIP as the key autophagy receptor in TOM1-mediated degradation of the SARS-CoV-2 E protein. Notably, the TOM1-TOLLIP-E axis operates independently of ubiquitination, distinguishing it from canonical ubiquitin-dependent receptor pathways. This ubiquitin-independent mechanism underscores the versatility of autophagy in recognizing viral proteins and suggests that SARS-CoV-2 E protein is subject to multiple host quality-control pathways, including both proteasomal and autophagic degradation.

We observed that TOM1 binds both the SARS-CoV-2 E and M proteins, but it selectively promotes the degradation of the E protein. This selectivity may be due to TOM1’s inability to efficiently deliver the M protein to autophagosomes. Similar cargo selectivity has been reported for NBR1 and SQSTM, which bind multiple viral proteins but selectively degrade only certain targets (25, 26). The SARS-CoV-2 E protein is an essential structural component that contributes to virion assembly, ion channel activity, and pathogenic inflammation (50–53). Previous work demonstrated that RNF5 and RNF185 promote proteasomal degradation of E protein, while our study provides the first evidence that autophagy also regulates its stability (54, 55). By placing TOM1 upstream of TOLLIP, our results highlight a distinct route for targeting E protein to autophagosomes, reinforcing the view that E protein turnover is tightly controlled by redundant host pathways. This redundancy may reflect strong selective pressure on the virus to maintain E protein integrity and function.

While our data support a model in which TOM1 restricts SARS-CoV-2 replication by promoting autophagic degradation of the viral E protein, we acknowledge that the current evidence does not fully distinguish selective autophagy from a more general autophagic response, as this conclusion is primarily based on E protein degradation mediated by the TOM1-TOLLIP complex. Therefore, it remains unclear whether the TOM1-TOLLIP complex promotes E protein degradation indirectly by enhancing overall autophagic flux or instead mediates specific recognition and degradation of the E protein through the recruitment of additional selective autophagy receptors and cofactors. Furthermore, the molecular link between TOM1 and the autophagy machinery requires further clarification. Based on our findings and prior studies, we propose that TOM1 may act as a scaffold to facilitate the recruitment of TOLLIP to autophagosome, where viral proteins localize, thereby enhancing its coupling to the autophagy machinery. Given that TOLLIP possesses both ubiquitin-binding capacity and an LC3-interacting region, the TOM1-TOLLIP complex may function as a bridge linking viral cargo to autophagosomes. Alternatively, TOM1 may exert its antiviral effects primarily by regulating endosomal trafficking, thereby indirectly increasing the accessibility of viral proteins to the autophagic degradation pathway.

In summary, we uncover TOM1 as a novel antiviral factor that promotes autophagic degradation of the SARS-CoV-2 E protein and demonstrate that coronaviruses have evolved a conserved strategy to counteract this defense through NSP5-mediated cleavage. These findings advance our understanding of how coronaviruses exploit protease-substrate interactions to escape immune surveillance. From a therapeutic perspective, the availability of small-molecule inhibitors targeting NSP5 suggests that blocking NSP5 activity or disrupting its interaction with TOM1 may help preserve TOM1 stability and enhance its antiviral function. Although no compounds directly targeting TOM1 have been reported, structure-guided drug design coupled with high-throughput screening may facilitate the identification of inhibitors that interfere with the NSP5-TOM1 interface. In addition, targeting host pathways that stabilize TOM1 or enhance its expression may represent an alternative strategy to potentiate its antiviral activity. Furthermore, combination therapies integrating NSP5 inhibitors with modulators of autophagy could provide synergistic effects in restricting viral replication. Collectively, these results indicate that targeting the TOM1-NSP5 interface or enhancing TOM1 stability may represent promising therapeutic approaches against SARS-CoV-2 and related coronaviruses.

## MATERIALS AND METHODS

### Cells and viruses

HEK293T (ATCC, CRL-11268, Manassas, VA, USA), HEK293T-hACE2 (BEI Resources, NR-52511), HeLa (ATCC, CRM-CCL-2), and Vero (ATCC, CCL-81) were cultured as monolayers in Dulbecco’s modified Eagle’s medium (DMEM) (Hyclone, Logan, UT, USA) supplemented with 10% heat-inactivated (56°C, 30 min) fetal calf serum (FCS, GIBCO BRL, Grand Island, NY, USA) and maintained at 37°C with 5% CO_2_ in a humidified atmosphere. SARS-CoV-2 Omicron BA.5 (GenBank: PQ802753.1) viruses were propagated in Vero cells, and HEK293T-hACE2 cells in DMEM supplemented with 2% FCS and titered using the median tissue culture infectious dose (TCID_50_) assay (56). All experiments of infectious SARS-CoV-2 were conducted under Biosafety Level 3 facilities.

### Antibodies and reagents

Anti-MYC polyclonal antibody (pAb) (16286-1-AP) and anti-Flag pAb (20543-1-AP) were purchased from Proteintech Group. Monoclonal mouse anti-Flag (F1804) antibody was purchased from Sigma. Anti-HA pAb (71-5500) was obtained from Invitrogen. Mouse anti-ACTB/β-actin (A00702) mAb was purchased from GenScript. The rabbit anti-TOM1 (A9273) pAb, anti-ATG5 (A11427) pAb, anti-BECN1 (A7353) pAb, and anti-MAP1LC3B/LC3B (A21800) pAb, rabbit anti-pro-CASP3/caspase 3 (A19654), and anti-CASP3/caspase 3 (A19664) mAb were purchased from ABclonal Biotechnology. SARS-CoV-2 nucleocapsid (GTX635679) antibody was purchased from GeneTex. Mouse anti-GFP (ab1218) mAb was purchased from Abcam. Horseradish peroxidase (HRP)-conjugated goat anti-rabbit (111-035-045) or -mouse (115-035-062) secondary antibodies were purchased from Jackson Immunoresearch. Alexa Fluor 647-labeled goat anti-rabbit IgG (A32733), Alexa Fluor 488-labeled goat anti-mouse IgG (A11001), and Alexa Fluor 405-labeled goat anti-rabbit IgG (A31556) were obtained from Invitrogen. Chemical reagents, including TNF-α (E7641), vinblastine (S4504), bafilomycin A1 (S1413), 3-methyladenine (S2767), and MG132 (S2619), were purchased from Selleck. CHX (66-81-9) was obtained from Sigma. Z-VAD-FMK (HY-16658B), rapamycin (AY-22989), torin 1 (HY-13003), and Mpro inhibitor N3 hemihydrate (HY-136149A) were purchased from MedChemexpress.

### Plasmids and transfection

The plasmids encoding for TOM1 and TOLLIP were purchased from Wuhan Miaolingbio. The TOM1 mutants (Q340A, Q354A, and Q380A) were generated by site-directed mutagenesis. The truncated TOM1 mutants, Flag-TOM1-MYC, TOM1-N, and TOM1-C were cloned into the VR1012 vector. Eukaryotic expression plasmids encoding SARS-CoV-2 proteins (N, E, S, M, PLpro, and NSP5) were provided by Professor Wang Peihui (Shandong University). The NSP5 plasmids of MERS-CoV and SARS-CoV were purchased from Wuhan Miaolingbio. Their corresponding inactive NSP5 mutants were inserted into the VR1012 vector with a HA tag. All plasmid constructs were validated by DNA sequencing. Lipofectamine 2000 Reagent (Invitrogen, 11668-019) was used for transient transfection of DNA into Vero, HeLa, and HEK293T cells according to the manufacturer’s instructions. All oligonucleotide primers used in this study are listed in Table S1.

### shRNA construction

The shRNA of ATG5, BECN1, and TOLLIP with the following target site was inserted into the lentiviral vector pLKO.1-puro. shATG5: 5′- AATTCGCCTTTCATTCAGAAGCTGTTTCTCGAGAAACAGCTTCTGAATGAAAGGTTTTTTG-3′ and 5′- GATCCAAAAAACCTTTCATTCAGAAGCTGTTTCTCGAGAAACAGCTTCTGAATGAAAGGCG-3′. shBECN1: 5′- AATTCGCTCAAGTTCATGCTGACGAATCTCGAGATTCGTCAGCATGAACTTGAGTTTTTTG- 3′ and 5′- GATCCAAAAAACTCAAGTTCATGCTGACGAATCTCGAGATTCGTCAGCATGAACTTGAGCG-3′. shTOLLIP: 5′- AATTCGCGACTGAACATCACGGTGGTACTCGAGTACCACCGTGATGTTCAGTCGTTTTTTG-3′ and 5′- GATCCAAAAAACGACTGAACATCACGGTGGTACTCGAGTACCACCGTGATGTTCAGTCGCG-3′.

### CRISPR-Cas9-mediated TOM1 gene knockout

*TOM1-KO* Vero cells and HEK293T-hACE2 cells were constructed using the CRISPR/Cas9 system, as described previously (57). The single guide RNA (sgRNA) targeting the *TOM1* gene (sequence: 5′-GCGAGCGGCTGCTGAGCCGGG-3′) was designed using the online software (CCTop, https://cctop.cos.uni-heidelberg.de) and then inserted into the lentiCRISPR v2 vector. HEK293T cells were cultured in 10-cm dishes and co-transfected with lentiCRISPRv2-TOM1-sgRNA, pMD2.G (Addgene #12259), and psPAX2 (Addgene #12260). The supernatant containing lentivirus was collected 48 h post-transfection. Vero cells and HEK293T-hACE2 cells were seeded into twelve-well plates and cultured to approximately 30% confluence prior to infection; 48 h post-transfection, 2 μg/mL (HEK293T-hACE2) or 5 μg/mL (Vero cells) of puromycin was added for positive clone selection. Single cells were subsequently seeded into 96-well plates by serial dilutions to isolate single-cell colonies. These clones were expanded and screened for *TOM1* knockout by sequencing and western blotting analysis.

### RNA extraction and RT-qPCR

For real-time quantitative PCR (RT-qPCR), intracellular RNA and viral RNA were extracted from the indicated cell lines or supernatants using TRIzol reagent (Invitrogen, catalog no. 15596018CN). The RNA was subsequently reverse transcribed by a High-Capacity cDNA Reverse Transcription Kit (Applied Biosystems, catalog no. 4368814). RT-qPCR was conducted on an Mx3005P instrument with Power SYBR Green PCR Master Mix (ABI, Carlsbad, Cat. No. 4367659). Amplification of the target fragment was performed under the following conditions: pre-denaturation at 95°C for 2 min, then followed by 40 cycles of 95°C for 15 s, 57°C for 15 s, and 68°C for 20 s. Primer sequences for real-time PCR employed in this study are provided in Table S1.

### Immunofluorescence assay (IFA)

HeLa cells were seeded on glass-bottom culture plate dishes and transfected with the indicated plasmids. After transfection, the cells were fixed in 4% paraformaldehyde for 15 min, washed with PBS, permeabilized with 0.25% Triton X-100 at room temperature for 10 min, washed with PBS, blocked with 5% bovine serum albumin for 1 h at room temperature, and then incubated at room temperature for 2 h with the indicated primary antibodies. Following three washes with PBST (Sigma, P3563), the cells were subjected to incubation with Alexa Fluor-conjugated secondary antibodies for 1 h and washed with PBST; nuclei were counterstained with 4′,6-diamidino-2-phenylindole (DAPI, Sigma, D9542) for 5 min. Finally, the cells were examined using a Nikon laser confocal microscope.

### Western blotting assay

Cells were harvested after the indicated treatments and lysed in buffer containing 50 mM Tris-HCl (pH 7.8), 150 mM NaCl, 1.0% NP-40, 5% glycerol, and 4 mM EDTA. The resulting lysates were mixed with 1× sample loading buffer (0.08 M Tris, pH 6.8; 2.0% SDS; 10% glycerol; 0.1 M dithiothreitol; and 0.2% bromophenol blue) and heated at 95°C for 30 min to achieve complete protein denaturation. Equivalent amounts of protein were resolved by 12.5% SDS-polyacrylamide gel electrophoresis and transferred onto polyvinylidene fluoride (PVDF) membranes. After blocking with 5% skim milk, the membranes were incubated sequentially with primary antibodies followed by horseradish peroxidase-linked secondary antibodies. Chemiluminescent signals were visualized using an ultrasensitive ECL detection reagent (B500024, Proteintech), and band intensities were quantified with ImageJ software (58).

### Co-immunoprecipitation

After transfection with the corresponding plasmids, the cells were harvested in 1 mL lysis buffer (50 mM Tris at pH 7.5, 150 mM NaCl, 1% NP-40) supplemented with protease inhibitors at 4°C for 4 h, and subsequently clarified by centrifugation at 12,000 × *g* for 10 min at 4°C. The supernatant was mixed with antibody-conjugated protein G agarose beads and incubated overnight at 4°C. Following incubation, the beads were washed four times with washing buffer (20 mM Tris, pH 7.5, 100 mM NaCl, 0.1 mM EDTA, and 0.05% Tween 20) at 4°C, centrifuged at 800 × *g* for 1 min. Proteins dropped down by beads were eluted with 0.1 M glycine-HCl elution buffer (pH 2.5) and analyzed by immunoblotting (56, 58).

### Virus infection

HEK293T-hACE2 cells and Vero cells were transfected with an overexpression plasmid using Lipofectamine 2000 Reagent (Invitrogen, catalog no. 11668-019) and infected with Omicron BA.5 (MOI = 0.01) 24 h after transfection. After 2 h of incubation, HEK293T-hACE2 cells and Vero cells were cultured in Dulbecco’s modified Eagle’s medium containing 2% fetal bovine serum for 24 or 48 h. Cells and culture supernatants were collected at specific time points after infection. Viral titers were analyzed using the TCID_50_ assay. Cells were used for western blot analysis to assess viral protein expression.

### Cell viability assay

Cell viability was measured using the CCK-8 kit (TransGen Biotech, FC101-02) following the manufacturer’s protocol. Briefly, *TOM1 KO* or WT cells were seeded into 96-well plates, and viability was measured at 24 h and 48 h post-seeding. CCK-8 reagent (10 μL/well) was added, followed by incubation at 37°C for 1 h, and absorbance was recorded at 450 nm using a microplate reader.

### Mass spectrometry (MS)

HEK293T cells were transfected with SARS-CoV-2 NSP5-DM or VR1012 for 36 h and then subjected to MG132 treatment for 10 h before harvest. Protein enrichment was performed by co-immunoprecipitation, followed by MS analysis of the eluates. Mass spectrum analysis was performed by the National Center for Protein Science (Beijing, China).

### Statistical analysis

Data analyses were performed using GraphPad Prism. All results are based on three independent experiments and are presented as the mean ± standard deviation (SD). Statistical analyses were conducted using an unpaired, two-tailed Student’s *t*-test (NS, not significant; *, *P* < 0.05; **, *P* < 0.01; ***, *P* < 0.001; ****, *P* < 0.0001).

## Data Availability

Data will be made available on request.

## SUPPLEMENTAL MATERIAL

Supplemental material jvi.00434-26-s0001.docx Fig. S1 to S5; Table S1.

ASM does not own the copyrights to Supplemental Material that may be linked to, or accessed through, an article. The authors have granted ASM a non-exclusive, world-wide license to publish the Supplemental Material files. Please contact the corresponding author directly for reuse.
