# Supplementary material for: Cleavage of TOM1 by the SARS-CoV-2 main protease NSP5 prevents autophagic degradation of viral envelope
Source: J Virol. 2026 Jun 12;100(7):e00434-26. doi: 10.1128/jvi.00434-26 (PMC13386971; doi:10.1128/jvi.00434-26)
Supplement: Supplemental material — Fig. S1 to S5; Table S1. [file jvi.00434-26-s0001.docx]

**Cleavage of TOM1 by the** **SARS-CoV-2 main protease NSP5 prevents autophagic degradation of viral envelope**

Qingxiang Zhang^a^, Jingguo Xin^a^, Chunlei Wang^a^, Xue Zhang^b^, Yuan Gao^a^, Wenying Gao^a *^, Wenyan Zhang^a *^

^a^ Institute of Virology and AIDS Research, Centre of Infectious Diseases and Pathogen Biology, Key Laboratory of Organ Regeneration and Transplantation of the Ministry of Education, the First Hospital of Jilin University

^b^ Jilin Provincial Key Laboratory on Molecular and Chemical Genetics, the Second Hospital of Jilin University, Changchun, China

*Corresponding authors. Institute of Virology and AIDS Research, First Hospital of Jilin University, No.1, Xinmin Street, Changchun, Jilin, 130000, E-mail: [zhangwenyan@jlu.edu.cn](mailto:zhangwenyan@jlu.edu.cn); gaowenying@jlu.edu.cn.

This PDF file includes:
Supplemental Fig S1-5
Supplemental Table S1


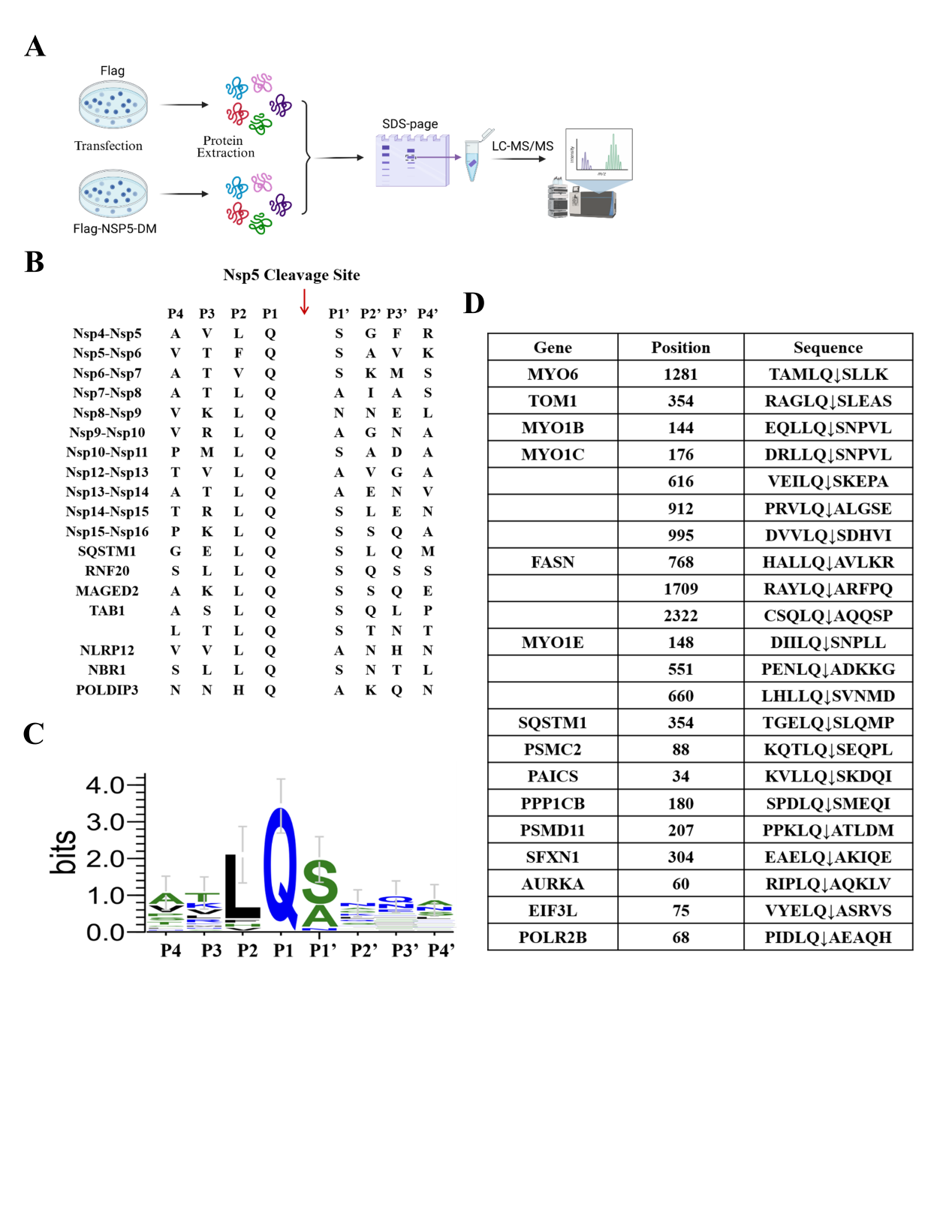


**Fig. S1. Screening of SARS-CoV-2 NSP5 Substrates.** (**A**) HEK293T cells were transfected with Flag-NSP5-DM for 24 h, cell lysates were incubated with protein G agarose beads with anti-Flag antibodies, followed samples were prepared for mass spectrum (MS). (**B**) Cleavage sequences of viral and host substrates targeted by the SARS-CoV-2 NSP5 protease were collected, and analyzed Glutamine (Q) at the P1 position as conserved site across the various substrates. (**C**) Sequence logo of the cleavage site was performed using WebLogo (https://weblogo.berkeley.edu/logo.cgi), based on the known SARS-CoV-2 NSP5 cleavage sites. (**D**) Candidate proteins containing the NSP5 cleavage motif.


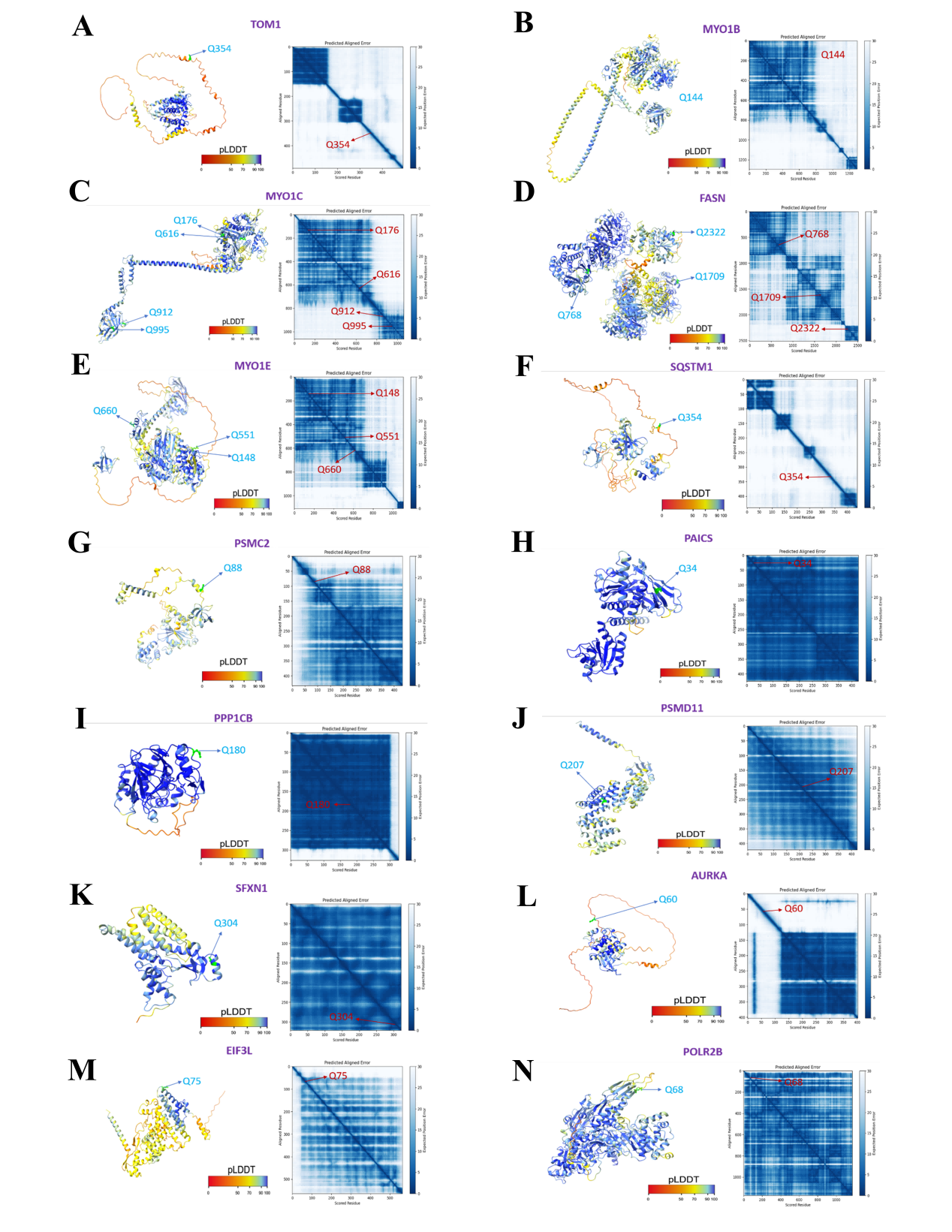


**Fig. S2. AlphaFold3 performs structural prediction for 14 substrate proteins.** (**A-N**) Panels A-N show the structural predictions and PAE matrices for TOM1, MYO1B, MYO1C, FASN, MYO1E, SQSTM1, PSMC2, PAICS, PPP1CB, PSMD11, SFXN1, AURKA, EIF3L, and POLR2B, respectively. In each panel, the left side depicts the AlphaFold3-predicted structures, color-coded according to pLDDT scores, with predicted cleavage sites highlighted in blue and the residues involved in cleavage emphasized. The right side displays the corresponding PAE matrices, which evaluate the confidence in the relative positioning of amino acid residues, where higher PAE values represent higher confidence.


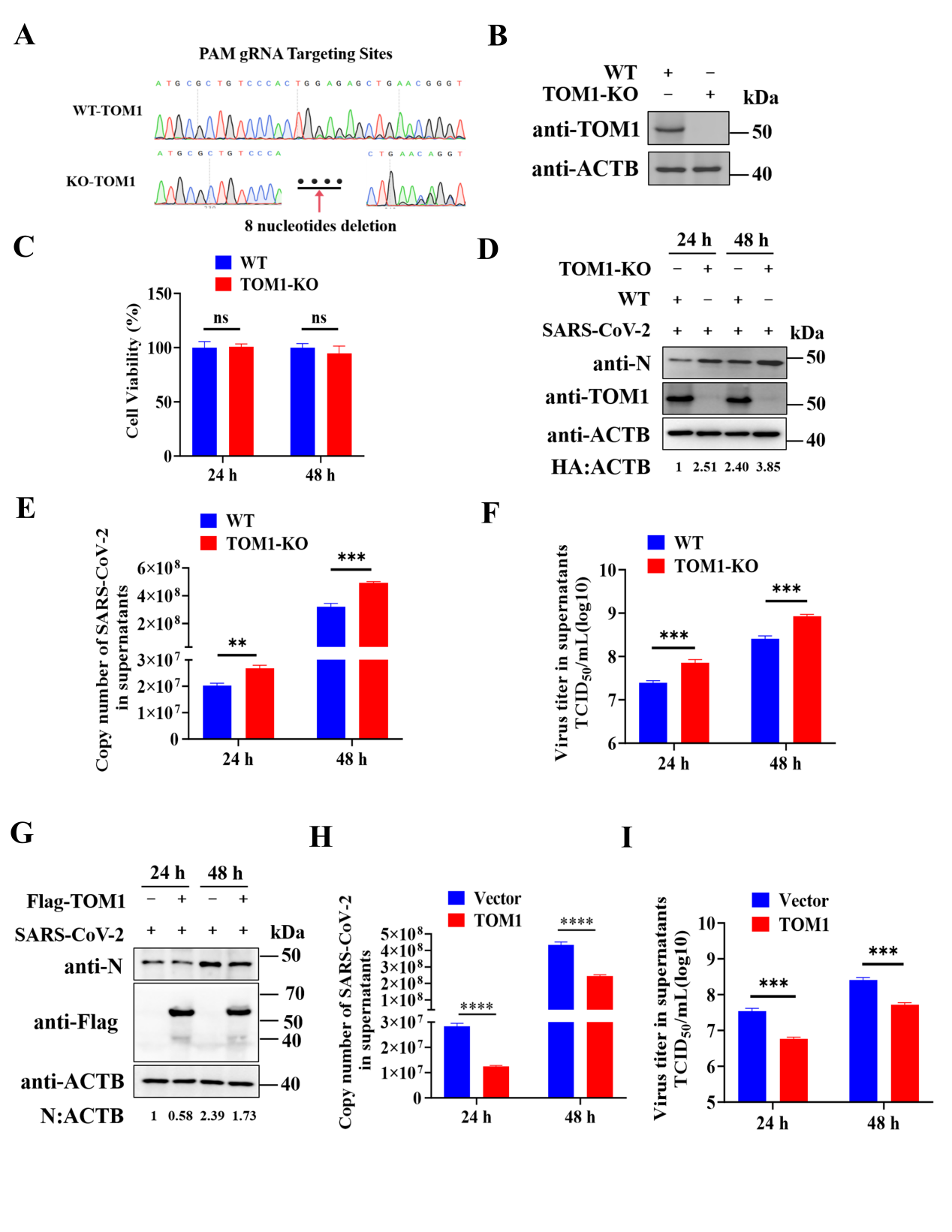


**Fig. S3. TOM1 inhibits SARS-CoV-2 replication in Vero cells.** (**A and B**) Validation of TOM1 knockout in Vero cells by DNA sequencing (A) and IB analysis (B). (**C**) Cell viability of WT and TOM1-KO Vero cell lines. (**D-F**) WT and TOM1-KO Vero cell lines were infected with SARS-CoV-2, the cells were collected at the indicated times for IB (D), the harvested supernatants were used for RT-qPCR (E) and TCID_50_ (F) assays. (**G-I**) Vero cells were transfected with Flag-TOM1 or an empty vector followed by SARS-CoV-2 infection. At 24 and 48 hpi, the cells were collected for IB (G), the harvested supernatants were used for RT-qPCR (H) and TCID_50_ (I) assays. Data are presented as mean ± SEM from three independent experiments.(ns, not significant; *P < 0.05; **P < 0.01; ***P < 0.001; ****P < 0.0001).


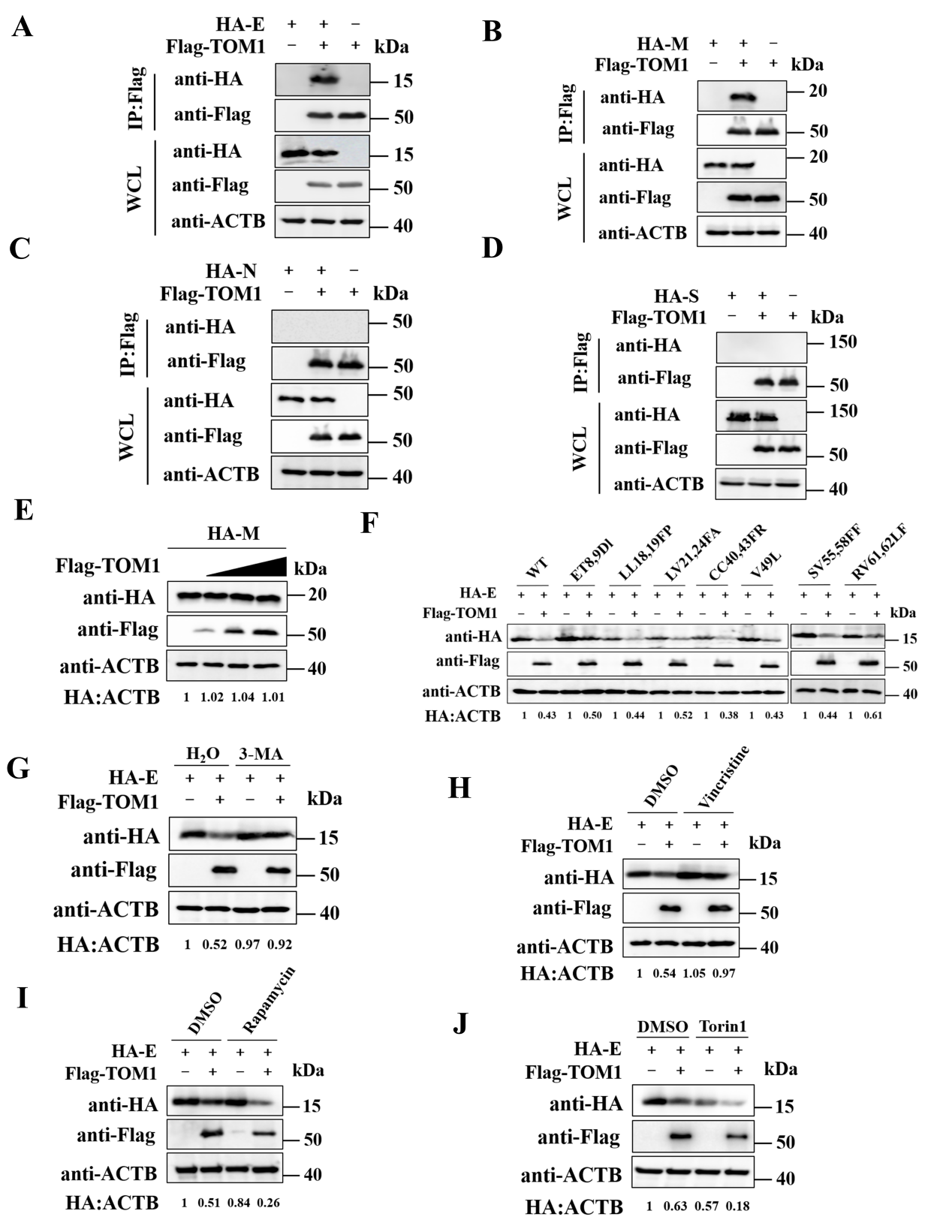


**Fig. S4. TOM1 targets SARS-CoV-2 E protein for autophagic degradation.** (**A-D**) HEK293T cells were co-transfected with Flag-TOM1 and either HA-tagged S, E, M, or N protein, then added 10 µM Bafilomycin A1 for 12 h to avoid the E degradation prior to harvest. After 48 h, cell lysates were co-IP using anti-Flag antibody, followed by IB analysis. (**E**) HEK293T cells were transfected with increasing amounts of Flag-TOM1 together with HA-M protein, followed IB analysis. (**F**) HEK293T cells were co-transfected with TOM1 and E alleles from different SARS-CoV-2 variants for 28 h, followed IB analysis. (**G-J**) HEK293T cells were co-transfected with Flag-TOM1 and HA-E plasmids, subsequently treated with 3-MA (10 mM) (G), Vincristine (50 µM) (H), Rapamycin (50 nM) (I) or Torin1 (200 nM) (J) for 12 h before harvesting, then cell lysates were analyzed by IB.


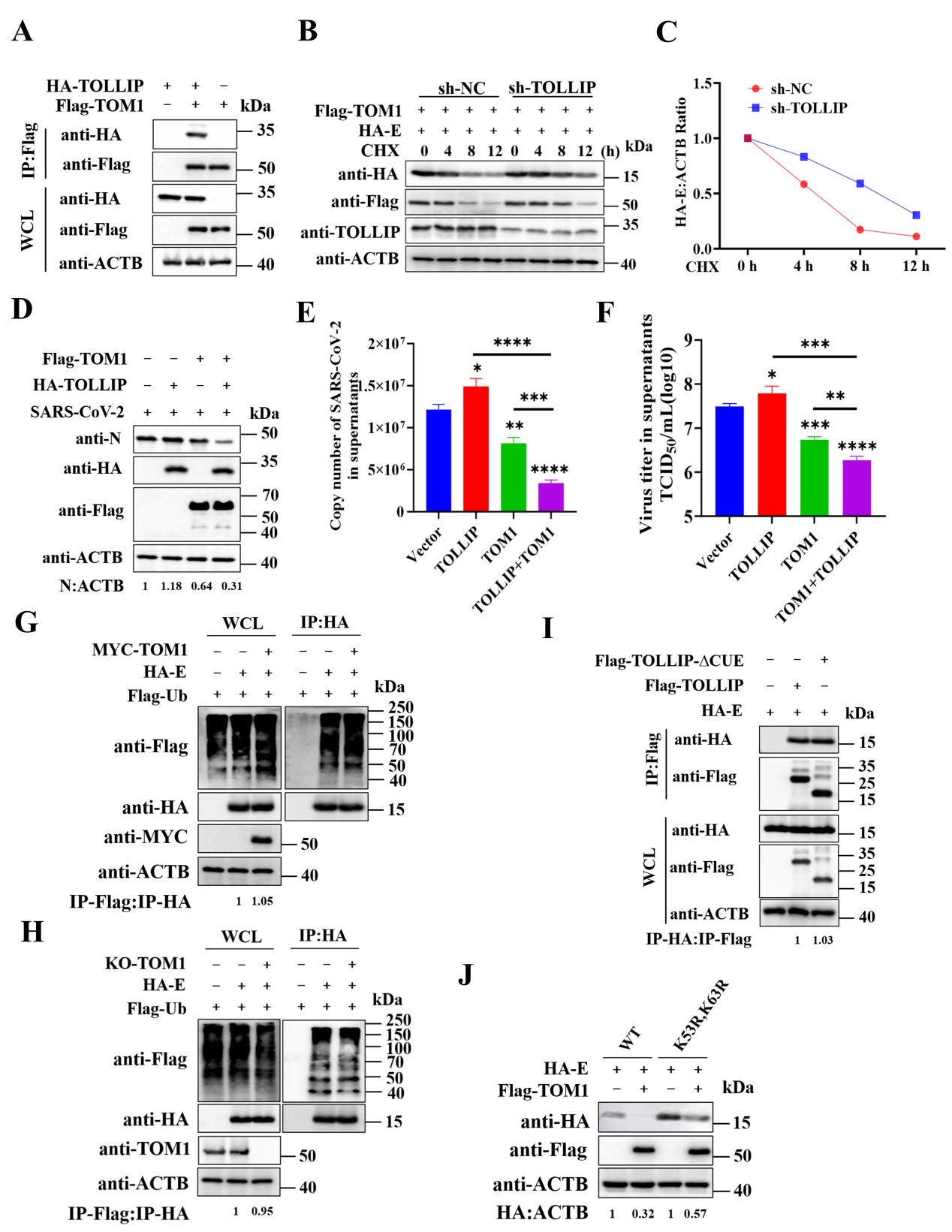


**Fig. S5. TOM1 recruits TOLLIP as an autophagy receptor to facilitate the degradation of the E protein.** (**A**) HEK293T cells were co-transfected with Flag-TOM1 and HA-TOLLIP. After 48 h, cell lysates were co-IP using anti-Flag antibody, followed by IB analysis. (**B**) HEK293T cells were co-transfected with Flag-TOM1, HA-E, and scramble or shTOLLIP for 24 h, then the cells were collected at the indicated time points to measure E protein levels by IB after 100 ug/mL CHX treatment. (**C**) Quantification of E protein levels from panel G, normalized to ACTB. **(D-F**) HEK293T-hACE2 cells were transfected with Flag-TOM1, HA-TOLLIP or both followed by SARS-CoV-2 infection. At 24 hpi, the cells were collected for IB (D), the harvested supernatants were used for RT-qPCR (E) and TCID_50_ (F) assays. Data are presented as mean ± SEM from three independent experiments. (ns, no significant; *P < 0.05; **P < 0.01; ***P < 0.001; ****P < 0.0001). (**G**) HEK293T cells were co-transfected with MYC-TOM1, HA-E and Flag-Ub, then added 10 µM Bafilomycin A1 for 12 h to avoid the E degradation prior to harvest. After 48 h, cell lysates were co-IP using anti-HA antibody, followed by IB analysis. (**H**) WT and TOM1-KO HEK293T cell lines were co-transfected with Flag-Ub and HA-E, then added 10 µM Bafilomycin A1 for 12 h to avoid the E degradation prior to harvest. After 48 h, cell lysates were co-IP using anti-HA antibody, followed by IB analysis. (**I**) HEK293T cells were co-transfected with HA-E and either Flag-tagged TOLLIP or TOLLIP-∆CUE plasmid, then added 10 µM Bafilomycin A1 for 12 h to avoid the E degradation prior to harvest. After 48 h, cell lysates were co-IP using anti-Flag antibody, followed by IB analysis. (**J**) HEK293T cells were transfected with Flag-TOM1 and HA-E or HA-E^K53R,K63R^ for 28 h. IB analysis of protein with the indicated antibodies.

Table S1. Primers used for plasmid construction and RT-qPCR in this study

| Name | Forward sequence (5ʹ-3ʹ) | Reverse sequence (5ʹ-3ʹ) |
| --- | --- | --- |
| *TOM1^Q340A^* | caacctctcatccGCgctggcaggaatgaacctg | tgccagcGCggatgagaggttgccggtggc |
| *TOM1^Q354A^* | tgtgagagctggcctgcagtctctggaggcctc | ctgcaggccagctctcacactgctggagcc |
| *TOM1^Q380A^* | gcagctcactggctgacGCacggaaagaggtaaa | tGCgtcagccagtgagctgccccgtgtcagcgcaaa |
| *TOM1-N* | AGTCACCGTCGTCGACatggactttctcctggggaaccc | CACAGCAGATCTGGATCCctgcaggccagctctcacactg |
| *TOM1-C* | AGTCACCGTCGTCGACtctctggaggcctctggtcgact | CACAGCAGATCTGGATCCtcataaggcaaacagcatgtcat |
| *TOM1(181-492)* | GGATGACGACGATAAGGAATTCctggagttccc | GAATTCCTTATCGTCGTCATCCTTGTAATCCATGGTGG |
| *TOM1(Δ181-312)* | atgaggacctgcggaggaaaggcaaggccccaagtga | gcctttcctccgcaggtcctcatagatggtgacca |
| *TOM1(1-312)* | ggttccgaacaggccagaccaccCTCGAGCTAGC | ggtggtctggcctgttcggaaccgttcaaac |
| *TOM1(1-180)* | GATGACGACGATAAGGAATTCCTCGAGCTAGCAGA | GAATTCCTTATCGTCGTCATCCTTGTAATCCATGG |
| *TOM1(313-492)* | GACGACGATAAGGAATTCaaggccccaagtgagg | GAATTCCTTATCGTCGTCATCCTTGTAATCCATG |
| *TOLLIPΔCUE* | gcatggtgcccgtggccctgcccCTCGAGCTAGC | AGgggcagggccacgggcaccatgccgg |
| *SARS-CoV-2-NSP5^H41A^* | GGTCTACTGCCCTAGAGCCGTGATCTGCACCAG | GGCTCTAGGGCAGTAGACCACATCGTCCAGCCAC |
| *SARS-CoV-2-NSP5^C145A^* | TTTTCTGAACGGCTCTGCTGGCAGCGTGGGCT | AGCAGAGCCGTTCAGAAAACTGCCCTTGATTGTG |
| *SARS-CoV-NSP5^H41A^* | AGTGTACTGCCCCAGAGCCGTGATTTGTACCGCCG | GGCTCTGGGGCAGTACACTGTGTCGTCCAGCCACAG |
| *SARS-CoV-NSP5-C145A* | CTTTCTGAACGGATCCGCCGGAAGCGTGGGCTTTA | GGCGGATCCGTTCAGAAAGCTGCCCTTGATGGT |
| *MERS-CoV-NSP5^H41A^* | gtctggtgcccacgaGCcgtaatgtgcccggctg | cgGCtcgtgggcaccagactgtgttgtcaagccaa |
| *MERS-CoV-NSP5^C145A^* | taagggttcctttctgGCtggttcttgtggtagtg | aGCcagaaaggaacccttaattgtgtagttaggg |
| *qPCR-TOM1* | atgctggctctcacagtctt | accagctccgtcagcatctc |
| *qPCR-GAPDH* | CCCATCACCATCTTCCAGG | TTCTCCATGGTGGTGAAGAC |
| *qPCR-SARS-CoV-2-N* | CAGACATTTTGCTCTCAAGCTG | TTGCTGCTGCTTGACAGATT |
| *qPCR-SARS-CoV-2-E* | CGATCTCTTGTAGATCTGTTCTC | ATATTGCATTGCAGCAGTACGCACA |
